# Multi-Trait Body Shape Phenotypes and Breast Cancer Risk in Postmenopausal Women: A Causal Mediation Analysis in the UK Biobank Cohort

**DOI:** 10.1007/s44197-024-00226-4

**Published:** 2024-04-10

**Authors:** Amina Amadou, Heinz Freisling, Anja M. Sedlmeier, Patricia Bohmann, Emma Fontvieille, Andrea Weber, Julian Konzok, Michael J. Stein, Laia Peruchet-Noray, Anna Jansana, Hwayoung Noh, Mathilde His, Quan Gan, Hansjörg Baurecht, Béatrice Fervers

**Affiliations:** 1 Department of Prevention Cancer Environment, Centre Léon Bérard, 28 rue Laënnec, Lyon Cedex 08, 69373 France; 2 Inserm U1296 Radiations : Défense, Santé, Environnement, Lyon, France; 3 Nutrition and Metabolism Branch, International Agency for Research on Cancer (IARC), Lyon, France; 4 Department of Epidemiology and Preventive Medicine, University of Regensburg, Regensburg, Germany

**Keywords:** Breast cancer, Anthropometry, Body shape, Biomarker, Mediation, Interaction

## Abstract

Body shape phenotypes combining multiple anthropometric traits have been linked to postmenopausal breast cancer (BC). However, underlying biological pathways remain poorly understood. This study investigated to what extent the associations of body shapes with postmenopausal BC risk is mediated by biochemical markers. The study included 176,686 postmenopausal women from UK Biobank. Four body shape phenotypes were derived from principal component (PC) analysis of height, weight, body mass index, waist and hip circumferences, and waist-to-hip ratio (WHR). The four-way decomposition of the total effect was used to estimate mediation and interaction effects simultaneously as well as the mediated proportions. After 10.9 years median follow-up, 6,396 incident postmenopausal BC were diagnosed. There was strong evidence of positive associations between PC1 (general obesity) and PC2 (tall, low WHR), and BC risk. The association of PC1 with BC risk was positively mediated by testosterone and negatively by insulin-like growth factor-1 (IGF-1), with the overall proportion mediated (sum of the mediated interaction and pure indirect effect (PIE)) accounting for 11.4% (95% confidence intervals: 5.1 to 17.8%) and -12.2% (-20.5% to -4.0%) of the total effect, respectively. Small proportions of the association between PC2 and BC were mediated by IGF-1 (PIE: 2.8% (0.6 to 4.9%)), and sex hormone-binding globulin (SHBG) (PIE: -6.1% (-10.9% to -1.3%)). Our findings are consistent with differential pathways linking different body shapes with BC risk, with a suggestive mediation through testosterone and IGF-1 in the relationship of a generally obese body shape and BC risk, while IGF-1 and SHBG may mediate a tall/lean body shape-BC risk association.

**Supplementary Information:**

The online version contains supplementary material available at 10.1007/s44197-024-00226-4.

## Introduction

Strong evidence links obesity (defined by high body mass index (BMI) ≥ 25 kg/m^2^); or indicators of body fat distribution, such as waist circumference (WC : men > 102 cm, women > 88 cm), hip circumference (HC), and waist-to-hip ratio (WHR : men > 0.90, women > 0.80)) with the risk of postmenopausal breast cancer (BC) [1–4], the most frequent cancer which represents an important public health problem in women [5–9].

Most studies investigated single anthropometric traits in relation to BC risk, which might not adequately capture the complexity of body morphology, specifically among women who are similar in one trait but differ in others [10]. To address this issue, Ried et al. were the first to apply a principal component analysis (PCA)-based approach to estimate principal components (PC) representing body shapes derived from BMI, height, weight, WC, HC, and WHR [11]. The four derived body shape phenotypes explained over 99% of the total variation in these anthropometric traits and were differently associated with several indicators of metabolic health (e.g., hormonal, metabolic, and inflammatory biomarkers) [11]. This PCA-based approach has been subsequently applied by our team to reflect associations of these body shapes with the risk of cancer in the European Prospective Investigation into Cancer and Nutrition cohort (EPIC) [10]. A generally obese body shape and a tall, lean body shape were both positively associated with postmenopausal breast cancer risk, while the other two body shapes were not associated with such risk [10].

The underlying biological and metabolic mechanisms linking obesity to BC are multiple and complex. Obesity has been strongly associated with several metabolic alterations, including deregulation of sex hormones, overexpression of pro-inflammatory cytokines, insulin resistance, hyperactivation of insulin-like growth factor (IGF) pathways, hypercholesterolemia, as well as excessive oxidative stress [5, 12–14]. Several of these biomarkers (such as serum sex hormone-binding globulin (SHBG), IGF-1, testosterone, C-reactive protein (CRP)) have also been associated with BC risk [15–17]. However, whether these biomarkers mediate the body shape-BC relationship is unknown. Such knowledge could help understand the impact of body shapes on BC risk and possibly identify biological pathways.

The main objective of the present study was to investigate to what extent the presumed associations between body shape phenotypes and postmenopausal BC risk are mediated by biomarkers of metabolic health. The candidate biomarkers were selected based on their implication in the development of BC, as well as their associations with obesity.

## Materials and Methods

### Study Population

UK Biobank (http://www.ukbiobank.ac.uk/) is a prospective cohort study that recruited a total of 502,418 men and women, aged between 39 and 71 years at enrollment between 2006 and 2010. Study design and methodology have been described elsewhere [9, 18]. At the initial assessment center visit, participants completed a self-administered touchscreen questionnaire that included information on health, demographic, anthropometric, lifestyle, and medical history data, collected in 22 centers across England, Wales, and Scotland. Biological samples including blood, saliva, and urine were also collected at enrollment. The UK Biobank study was approved by the North West Multi-Center Research Ethics Committee, the National Information Governance Board for Health and Social Care in England and Wales, and the Community Health Index Advisory Group in Scotland (http://www.ukbiobank.ac.uk/ethics/). All participants provided written informed consent.

For the present study, we only included women, who were postmenopausal at the time of enrollment. Women were categorized as postmenopausal if they reported “yes” to the question “Have you had your menopause (periods stopped at least one year before enrollment)”, if they were older than 55 years [19] or reported a bilateral oophorectomy. Among these, we excluded women with prevalent cancer, those with missing or implausible anthropometry data, and with missing biomarker data. The study participants flowchart is given in *Supplementary Fig. *1. After exclusions, the analysis involved 176,686 postmenopausal women.

### Ascertainment of Breast Cancer Cases

Data on cancer diagnoses were provided by National Health Service (NHS) Digital and Public Health England for participants from England and Wales and by NHS Central Register (NHSCR) for participants residing in Scotland, and BC cases were ascertained through cancer registries [20]. For the present study, complete follow-up data were available up to 29 February 2020 for England and Wales; and 31 January 2021 for Scotland. All registrations coded as C50 using the 10th Revision of the International Classification of Diseases (ICD-10) were considered as invasive BC cases.

### Assessment of Anthropometric Measures

Height, weight, WC, and HC were assessed by trained personnel during the baseline assessment center visit [21]. Body weight (kilograms, kg) was measured using a Tanita BC418MA body composition analyzer. Height was measured using a Seca 240 cm height measure, while HC and WC measurements (cm) were assessed using a Seca 200 cm tape measure. BMI was calculated as body weight (kg) divided by height in meters squared (kg/m^2^), and WHR was calculated as WC divided by HC.

### Biomarker Assays

UK Biobank measures a wide range of biochemical markers from biological samples collected at baseline in all participants [22]. The biomarkers selected for the assay have been chosen because they are established risk factors for several diseases [22]. The present study examined biomarkers of metabolic health comprising markers of glucose (glucose, glycated hemoglobin, HbA1c, mmol/mol), insulin metabolism (IGF-1, nmol/L), inflammation (CRP, mg/L), sex hormones (testosterone and SHBG, nmol/L), blood lipids (triglycerides, HDL-cholesterol and cholesterol, mmol/L), as well as total protein (g/L). These biomarkers were selected based on their potential links with overweight/obesity, and BC risk [1, 12, 23]. We further explored other biomarkers of metabolic health that were moderately correlated to body shape phenotypes, to identify novel biomarkers that could influence their association with BC risk. These biomarkers included albumin (g/L), glucose (mmol/L), alanine amino-transferase (U/L), apolipoproteins A and B (g/L), cystatin C (mg/L), Gamma glutamyltransferase (U/L), total bilirubin (umol/L), and urate (umol/L).

The following biomarkers were measured with Beckman Coulter AU5800 analyzer (Beckman Coulter (UK), Ltd.) as part of the UK Biobank biomarker project [16, 17]. Triglycerides were quantified by Group Purchasing Organisation-Physician Owned Distributor (GPO-POD) analysis, cholesterol by cholesterol oxidase-peroxidase (CHOD-POD) method, HDL-cholesterol by enzyme immune-inhibition analysis, CRP by immunoturbidimetric-high sensitivity analysis, and total protein by Biuret analysis. Albumin was measured spectrophotometrically using bromocresol green, alanine amino-transferase and gamma glutamyltransferase by enzymatic rate, and glucose by hexokinase. Apolipoproteins A and B, and cystatin were quantified by immuno-turbidimetry, total bilirubin by colorimetric assay, and urate by uricase-PAP. Serum levels of HbA1c were measured by high-performance liquid chromatography analysis on a Bio-Rad, VARIANT II Turbo, and IGF-1 was quantified by chemiluminescence immunoassay (CLIA) technique (DiaSorin Ltd LIASON XL). SHBG was measured using the two-step competitive analysis method (Beckman Coulter, Unicel DxI 800), while testosterone was measured with a one-step competitive analysis (Beckman Coulter, Unicel DxI 800).

### **Statistical Analysis**

PCA was applied to the standardized residuals of height, weight, BMI, WC, HC, and WHR. BMI and WHR were included in the PCA, because these composite variables usually still show some correlation with weight and height, and WC and HC, respectively. The residuals were predicted from a separate regression of the six anthropometric traits with age and study center. From the PCA, we retained the first four PCs that explained 99% of the variation and represented orthogonal linear combinations of the six anthropometric traits [11]. Each component represented a weighted sum of the six transformed anthropometric traits and is independent of the other components. The weights of each trait per PC are referred to as loadings. We used “https://bodyvisualizer.com/” to visualize the four body shapes by computing the mean values of the six anthropometric traits among participants in the 95% and 5% percentiles of each PC. Pearson correlation coefficients were used to assess the correlations between the six anthropometric traits and PCs.

Cox proportional hazard regression was used to estimate the hazard ratios (HR) and corresponding 95% confidence intervals (CI) of the associations between each body shape PC (continuous and quintiles), and each biomarker (continuous) with BC risk. Continuous models for an increment of one standard deviation (SD) of each PC and biomarker were estimated. Age at entry was age at recruitment, and exit time was considered one of following: age at diagnosis of first incident BC, age of diagnosis of another cancer except non-melanoma skin cancer, age at end of follow-up, age at loss-to-follow-up, or age at time of death, whichever occurred first. The proportional hazards assumptions were tested using scaled Schoenfeld residuals. The shape of the exposure-response curve between each PC and BC risk was estimated using restricted cubic splines [24], with five knots placed at the 5th, 27.5th, 50th, 72.5th and 95th percentiles, as recommended by Harrell et al. for larger datasets [25]. Linear regression was performed to assess the associations between each PC and distinct biomarkers of metabolic health.

We employed med4way mediation analysis [26] to investigate whether metabolic biomarkers can act as individual mediators on the pathway between body shapes and postmenopausal BC risk. Med4way uses parametric regression models to estimate the components of the four-way decomposition of the total effect of the exposure (here: PC) on the outcome (BC) in the presence of the mediator (each biomarker of metabolic health) with which the exposure may interact. The total effect (TE) is decomposed into four components, i.e. the controlled direct effect (CDE, i.e. the effect of PC on BC neither due to mediation nor to interaction), the reference interaction effect (INTref, i.e. the effect due to interaction only), the mediated interaction effect (INTmed, i.e. due to both mediation and interaction) and the pure indirect effect (PIE, i.e. only due to mediation, but not interaction) [26, 27]. The CDE was estimated at a fixed level of the mediator (here: median). Two regression models were fitted: a Cox model for the outcome, and a linear regression model for the mediator. The variable for the interaction between the exposure and the mediator was automatically generated and added to the model for the outcome. In addition to the four components of the TE, we further estimated the proportions of the effect due to each component, including the proportion due to the CDE, the proportion due to the PIE, the proportion due to the INTref, the proportion due to the INTmed, as well as the overall proportion mediated (PIE + INTmed).

The crude models were stratified by age at recruitment in 5-year categories, and study center. All multivariable models were adjusted for the following potential confounders, identified by a directed acyclic graph (*Supplementary Fig. *2): age at recruitment, study center, healthy diet score, alcohol intake, smoking status, ethnicity, use of oral contraceptives, use of menopausal hormone treatment (MHT), physical activity, qualifications, Townsend deprivation index, and sedentary behavior. Healthy diet score was calculated based on consumption of these commonly food groups (fruits, vegetables, fish, processed meats, unprocessed red meats, whole grains, and refined grains) [28]. Sedentary behavior is the sum of time spent watching TV, time spent using the computer and time spent driving. Covariates, except for physical activity (missing values = 24.3%) and sedentary behavior (missing values = 4.2%), had less than 2% missing data. The multivariable analyses were thus conducted in the complete-case dataset, excluding all women with a missing value (*n* = 47,319) for any of the adjusted covariates, which resulted in a final sample size of 129,367 participants. In the mediation analysis, additional mutual adjustment for each biomarker was performed, by adjusting each mediator model for all other biomarkers.

The following sensitivity analyses were performed to assess the robustness of the main results: First, we excluded participants with less than two years of follow-up to control for potential reverse causation. Second, to account for the uncertainty of missing data in the adjustment variables, we performed multivariate imputation using chained equations (MICE) (‘mi impute’ in STATA) [29]. We used 10 iterations to impute multiple variables iteratively with fully conditional specification of prediction equations [29]. Variables with missing values were physical activity, alcohol intake, smoking status, use of oral contraceptives, use of MHT, qualifications, Townsend deprivation index and sedentary behavior. Third, we restricted the analyses to postmenopausal women, who answered having their periods stopped at least one year before enrollment, after exclusion of women, who were 55 years or older at enrollment, or reported a bilateral oophorectomy (*n* = 108,754). Finally, we investigated associations of single anthropometry measures (BMI, WC, WHR, and height) with BC risk. As sensitivity mediation analyses, additional models without mutual biomarkers adjustment were conducted. Additional mediation analyses were also conducted for BMI, WC, WHR, and height.

Body shape phenotypes from PC analysis was done with the package “FactoMineR” using R version 4.2.3, all other statistical analyses were performed using STATA 14.

## Results

### Characteristics of the Study Population

**Fig. 1:**
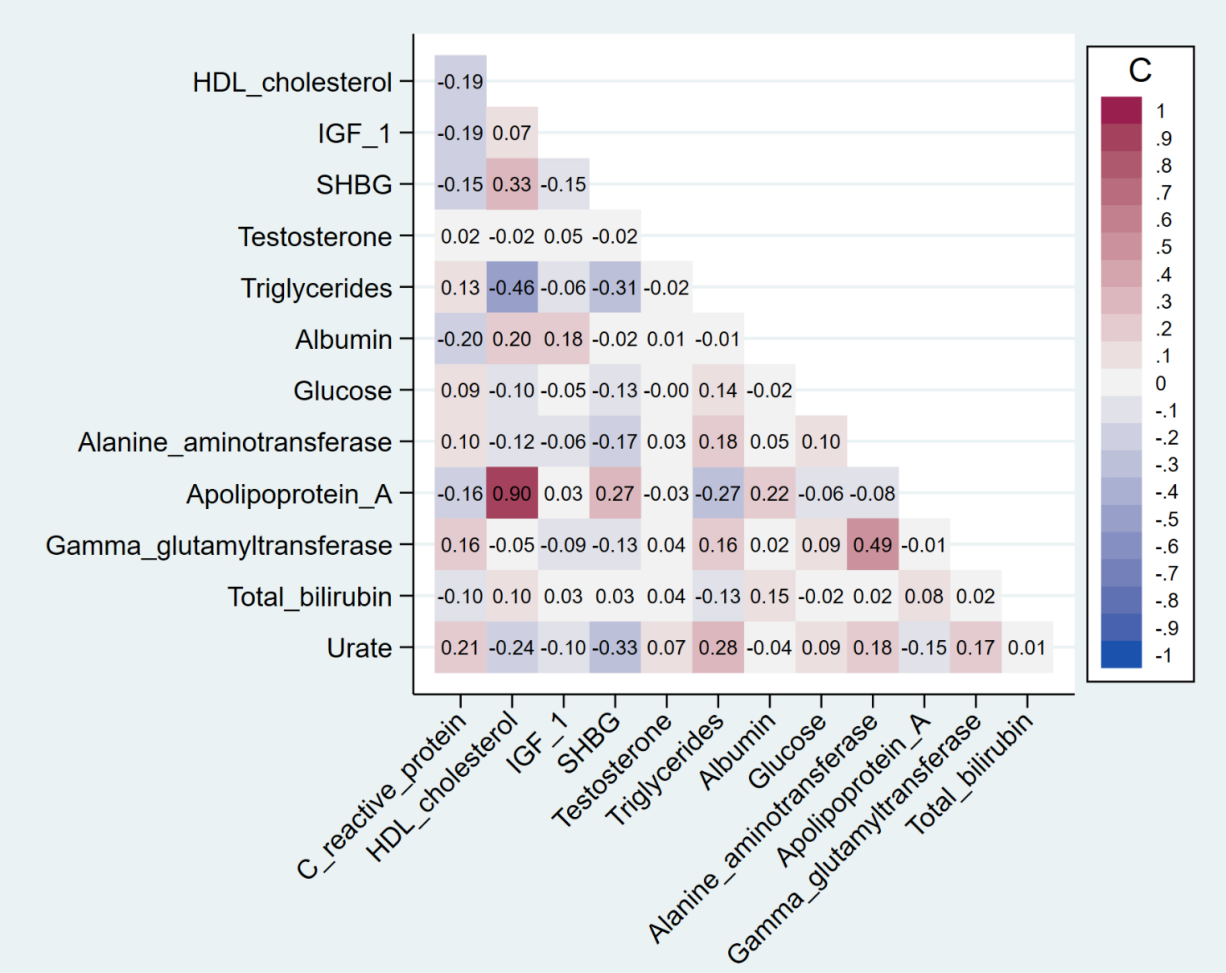
Pearson’s correlation matrix between the biomarkers. C coefficients of correlation, HDL cholesterol high-density lipoprotein cholesterol, IGF-1 insulin-like growth factor, SHBG sex hormone-binding globulin

After a median follow-up of 10.9 years (interquartile range = 10.1–11.7), 6,396 incident BC events were diagnosed among the 176,686 postmenopausal women. The characteristics of the study participants by cases/non-cases status are shown in *Supplementary Table*1. The average age at recruitment (± SD) was 61.0 (± 5.3) years for women with BC and 60.4 (± 5.7) years for women without BC. Compared to women without BC, participants with BC were more likely to have higher anthropometric measures, to be less physically active, to have a higher level of alcohol intake, and to be MHT users. The distribution of other characteristics was generally similar. Concentration levels of CRP, testosterone, and urate were slightly higher among women with BC compared to those without, while SHBG was lower. All other biomarkers’ concentration levels were comparable (*Supplementary Table*2). Overall, except between HDL-cholesterol and apolipoprotein A (correlation coefficient = 0.9), there were no strong correlations between biomarkers (Fig. 1).

### Body Shape Phenotypes

Loadings and explained variance of the six PCs for women are presented in *Supplementary Table*3. PC1 (65.7% of the total variation) described individuals with general obesity vs. a lean body shape (*Supplementary Fig. *3). PC2 (19.1% of the total variation) characterized tall individuals with low WHR vs. short individuals with large WHR (*Supplementary Fig. *4). PC3 (13.5% of the total variation) characterized tall individuals with high WHR vs. short individuals with low WHR (*Supplementary Fig. *5). PC4 (1.6% of the total variation) showed high loadings for BMI and weight, and low loadings for HC and WC (*Supplementary Fig. *6). Pearson ’s correlation coefficients between the six anthropometric measures and PCs were comparable to results with the loadings for the individual PCs (*Supplementary Fig. *7). Baseline characteristics of the study participants are further presented by quintiles of PC1 scores (Table 1). As compared to women in the lower quintile (Q1), women in the upper quintile (Q5) had higher anthropometric measures, except for height. Women in the two lowest quintiles of PC1 had a healthier diet, a higher educational level, a higher level of alcohol intake, a lower Townsend deprivation index, were more physically active, less sedentary, and more likely to be never smokers compared with those in the upper quintile of PC1.

**Table 1:** Characteristics of participants according to quintiles of scores of principal component 1 (general obesity) in the UK Biobank cohort study

	Quintile 1	Quintile 2	Quintile 3	Quintile 4	Quintile 5
N (%)	35,338 (20)	35,337 (20)	35,336 (20)	35,338 (20)	35,337 (20)
Follow-up time (years), median (IQR)	10.9 (10.1–11.7)	10.9 (10.1–11.7)	10.9 (10.1–11.7)	10.9 (10.1–11.7)	10.9 (10.0-11.6)
Age at recruitment (years), mean (SD)	60.4 (5.6)	60.4 (5.6)	60.6 (5.6)	60.7 (5.6)	60.0 (5.8)
Age at menarche (years), mean (SD)	13.1 (1.6)	13.0 (1.6)	13.0 (1.6)	12.9 (1.6)	12.7 (1.7)
Townsend deprivation index, mean (SD)	-1.7 (2.8)	-1.7 (2.8)	-1.6 (2.9)	-1.4 (3.0)	-0.78 (3.2)
Sedentary behavior (hours/day), mean (SD)	3.9 (1.9)	4.2 (1.9)	4.5 (2.0)	4.7 (2.1)	5.1 (2.3)
BMI (kg/m²), mean (SD)	21.8 (1.7)	24.4 (1.5)	26.4 (1.6)	29.0 (1.9)	34.9 (4.4)
Weight (kg), mean (SD)	56.4 (4.8)	63.8 (4.1)	69.2 (4.3)	76.1 (5.0)	91.9 (11.6)
Height (m), mean (SD)	1.61 (0.1)	1.62 (0.1)	1.62 (0.1)	1.62 (0.1)	1.62 (0.1)
WHR, mean (SD)	0.76 (0.05)	0.80 (0.1)	0.82 (0.1)	0.85 (0.1)	0.88 (0.1)
Waist circumference (cm), mean (SD)	70.6 (4.1)	78.1 (3.2)	84.0 (3.4)	90.8 (4.0)	103.8 (8.7)
Hip circumference (cm), mean (SD)	92.9 (4.4)	98.2 (4.0)	102.0 (4.2)	106.8 (4.8)	118.0 (9.5)
*Physical activity, n (%)*					
Low	3,555 (12.9)	3,949 (14.3)	4,454 (16.5)	5,339 (20.3)	6,996 (27.8)
Moderate	11,425 (41.4)	11,909 (43.3)	12,071 (44.7)	11,537 (43.7)	1,081 (42.9)
High	12,624 (45.7)	11,658 (42.4)	10,477 (38.8)	9,498 (36.0)	7,377 (29.3)
*Alcohol drinking, n (%)*					
Daily or almost daily	7,570 (21.5)	6,913 (19.6)	6,397 (18.1)	5,451 (15.5)	3,621 (10.3)
3 to 4 times a week	7,868 (22.3)	8,172 (23.1)	7,508 (21.3)	6,619 (18.8)	4,978 (14.1)
1 to 2 times a week	8,370 (23.7)	8,951 (25.3)	9,159 (26.0)	9,089 (25.8)	8,318 (23.6)
1 to 3 times a month	3,785 (10.7)	3,969 (11.2)	4,275 (12.1)	4,620 (13.1)	5,380 (15.3)
Special occasions only	4,380 (12.4)	4,472 (12.7)	4,904 (13.9)	5,873 (16.5)	7,910 (22.4)
Never	3,314 (9.4)	2,813 (8.0)	3,031 (8.6)	3,615 (10.2)	5,056 (14.3)
*Smoking status, n (%)*					
Never	21,925 (62.3)	21,010 (59.7)	20,524 (58.4)	19,967 (56.8)	19,468 (55.4)
Previous	10,185 (28.9)	11,375 (32.3)	11,832 (33.6)	12,324 (35.1)	12,736 (36.3)
Current	3,112 (8.8)	2,801 (8.0)	2,808 (8.0)	2,848 (8.1)	2,909 (8.3)
*Qualification, n (%)*					
None of the above	5,921 (17.0)	6,581 (18.9)	7,424 (21.4)	8,377 (24.2)	8,962 (25.9)
College or University degree	11,957 (34.4)	10,806 (31.1)	9,539 (27.5)	8,613 (24.9)	7,753 (22.4)
A levels/AS levels or equivalent	4,161 (12.0)	3,849 (11.1)	3,842 (11.1)	3,517 (10.1)	3,415 (10.0)
O levels/GCSEs or equivalent	7,834 (22.5)	8,357 (24.1)	8,356 (24.1)	8,186 (23.6)	8,072 (23.4)
CSEs or equivalent	1,311 (3.8)	1,401 (4.0)	1,465 (4.2)	1,642 (4.7)	1,814 (5.3)
NVQ or HND or HNC or equivalent	1,234 (3.5)	1,507 (4.3)	1,637 (4.7)	1,826 (5.3)	2,144 (6.2)
Other professional	2,324 (6.7)	2,227 (6.4)	2,380 (6.9)	2,461 (7.1)	2,386 (6.9)
*Ever use of oral contraceptive, n (%)*					
No	7,554 (21.4)	7,209 (20.5)	7,556 (21.4)	7,938 (22.6)	8,026 (22.8)
Yes	27,662 (78.6)	28,028 (79.5)	27,660 (78.6)	27,237 (77.4)	27,151 (77.2)
*Ever use of menopausal hormone therapy, n (%)*					
No	18,173 (51.6)	17,147 (48.7)	17,061 (48.4)	16,734 (47.6)	17,756 (50.6)
Yes	17,057 (48.4)	18,075 (51.3)	18,158 (51.6)	18,439 (52.4)	17,362 (49.4)
*Healthy diet score, n (%)*					
0 (unhealthy)	19 (0.1)	14 (0.04)	21 (0.1)	35 (0.1)	49 (0.1)
1	214 (0.6)	201 (0.6)	230 (0.6)	289 (0.8)	448 (1.3)
2	1,219 (3.4)	1,339 (3.8)	1,442 (4.1)	1,618 (4.6)	2,294 (6.5)
3	6,288 (17.8)	7,004 (19.8)	7,534 (21.3)	8,150 (23.1)	9,032 (25.6)
4	16,539 (46.8)	16,964 (48.0)	16,875 (47.8)	16,557 (46.8)	15,633 (44.2)
5	9,462 (26.8)	8,599 (24.3)	8,176 (23.1)	7,712 (21.8)	7,018 (19.8)
6 (healthy)	1,597 (4.5)	1,216 (3.4)	1,058 (3.0)	977 (2.8)	863 (2.4)
*Screening mammogram, n (%)*					
No	1,890 (5.4)	1,788 (5.1)	1,696 (4.8)	1,799 (5.1)	2,291 (6.5)
Yes	33,408 (94.6)	33,495 (94.9)	33,578 (95.2)	33,467 (94.9)	32,965 (93.5)
*Ethnicity, n (%)*					
White	33,999 (96.5)	33,959 (96.5)	33,773 (95.9)	33,428 (95.0)	33,086 (94.0)
Mixed	156 (0.4)	160 (0.4)	145 (0.4)	200 (0.6)	184 (0.5)
Asian	396 (1.1)	490 (1.4)	601 (1.7)	625 (1.8)	515 (1.5)
Black	163 (0.5)	240 (0.7)	353 (1.0)	566 (1.6)	1,065 (3.0)
Chinese	263 (0.8)	107 (0.3)	80 (0.2)	45 (0.1)	14 (0.04)
Other ethnic group	260 (0.7)	249 (0.7)	251 (0.7)	329 (0.9)	333 (1.0)

Mean (Standard deviation) and Counts (Percentages) are presented for continuous and categorical variables, respectively

IQR: interquartile range, BMI: body mass index, WHR: waist-to-hip ratio, healthy diet score (from unhealthy to healthy) was calculated based on consumption of these commonly food groups (fruits, vegetables, fish, processed meats, unprocessed red meats, whole grains, and refined grains) [28], Sedentary behaviour = sum of time spent watching television, time spent using computer and time spent driving, Qualification : A: advanced, AS: advanced subsidiary, O: ordinary, GCSE: general certificate of secondary education, CSE: certificate of secondary education, NVQ: national vocational qualification, HND: higher national diploma, HNC: higher national certificate

### Body Shape Phenotypes and Breast Cancer Risk

Table 2 shows crude and multivariable-adjusted associations between the four body shapes and BC risk. After adjusting for measured confounders, each 1 SD increment in PC1 (i.e., more of a generally obese body shape) was associated with a 12% (95% CI: 9–16%) higher relative risk of BC. Similarly, each 1 SD increment in PC2 (i.e., more of a tall and lean body shape) was associated with an 8% (95% CI: 5–11%) higher relative risk of BC. The linearity of these associations was confirmed in the analyses by quintiles (Table 2) and visually using restricted cubic splines (*Supplementary Figs. *8 and 9). In contrast, neither PC3 (i.e. more of a tall and centrally overweight body shape) nor PC4 (i.e., an “athletic” body shape) were associated with BC risk (Table 2, *Supplementary Figs. *10 and 11).

**Table 2:** Associations between principal components (PC) of body shape and postmenopausal breast cancer risk

	PC1 (“general obesity”)					PC2 (“tall, low WHR”)		PC3 (“tall, high WHR”)		PC4 (“athletic”)		
		Cases/non cases	HR (95% CI)	Cases/non cases			HR (95% CI)	Cases/non cases	HR (95% CI)	Cases/non cases	HR (95% CI)	
**Model** ^**1**^												
Continuous (for an increment of 1 SD)		6,396/170,290	1.13 (1.10–1.16)		6,396/170,290		1.10 (1.07–1.13)	6,396/170,290	1.05 (1.03–1.08)	6,396/170,290	1.03 (1.00-1.05)	
*Quintiles*												
I		1,005/34,333	1 (ref)		1,093/34,245		1 (ref)	1,190/34,147	1 (ref)	1,259/34,079	1 (ref)	
II		1,253/34,084	1.24 (1.14–1.35)		1,248/34,091		1.14 (1.05–1.24)	1,258/34,081	1.09 (1.00-1.18)	1,252/34,085	1.03 (0.95–1.12)	
III		1,296/34,040	1.29 (1.19–1.40)		1,285/34,051		1.17 (1.08–1.27)	1,239/34,096	1.06 (0.98–1.15)	1,273/34,064	1.06 (0.98–1.14)	
IV		1,368/33,970	1.38 (1.27–1.50)		1,318/34,019		1.21 (1.11–1.31)	1,327/34,011	1.12 (1.04–1.21)	1,331/34,006	1.11 (1.03–1.20)	
V		1,474/33,863	1.54 (1.42–1.67)		1,452/33,884		1.32 (1.22–1.43)	1,382/33,955	1.14 (1.05–1.23)	1,281/34,056	1.05 (0.97–1.14)	
**Model** ^**2**^												
Continuous (for an increment of 1 SD)		4,654/124,709	1.12 (1.09–1.16)		4,654/124,709		1.08 (1.05–1.11)	4,654/124,709	1.03 (1.00-1.06)	4,654/124,709	1.02 (0.99–1.05)	
*Quintiles*												
I		767/26,075	1 (ref)		762/23,595		1 (ref)	844/24,497	1 (ref)	910/24,363	1 (ref)	
II		955/25,773	1.26 (1.14–1.38)		906/24,500		1.13 (1.03–1.25)	932/24,938	1.09 (1.00-1.20)	948/25,000	1.05 (0.96–1.15)	
III		958/25,181	1.29 (1.17–1.42)		920/25,085		1.11 (1.01–1.23)	907/25,131	1.04 (0.95–1.15)	919/25,299	1.01 (0.92–1.11)	
IV		1.006/24,419	1.42 (1.29–1.57)		992/25,558		1.17 (1.07–1.29)	980/25,119	1.10 (1.00-1.21)	961/25,115	1.08 (0.98–1.18)	
V		968/23,261	1.49 (1.34–1.64)		1.074/25,971		1.24 (1.13–1.37)	991/25,024	1.08 (0.98–1.18)	916/24,932	1.03 (0.94–1.13)	

Model ^1^: Hazard ratios from Cox proportional hazards regression using age as the underlying time metric. Models were stratified by age at recruitment in 5-year categories and study center. *N* = 176,686

Model ^2^ : Hazard ratios from Cox proportional hazards regression using age as the underlying time metric. Multivariable models were stratified by age at recruitment in 5-year categories, study center, and adjusted for alcohol intake, smoking status, ethnicity, oral contraceptive, menopausal hormone treatment, physical activity, healthy diet score, qualifications, Townsend deprivation index, and sedentary behavior. All four principal components were mutually adjusted. The multivariable-adjusted analyses were then conducted in the complete-case dataset, excluding all women with a missing value (*n* = 47,319) for any of the adjusted covariates, resulting in a final sample size of 129,367 participants

PC: principal component; SD: standard deviation. WHR: waist-to-hip ratio

Results after excluding participants with less than two years of follow-up remained comparable to the main findings (*Supplementary Table*4). Results after multiple imputation of missing values in covariates yielded similar results but the 95% CIs were narrower; for PC3, the HR slightly increased to 1.05 (95% CI: 1.02–1.08). Sensitivity analyses based exclusively on postmenopausal women, who answered having their periods stopped for at least one year at the time of recruitment showed similar results (*Supplementary Table*4). Finally, further sensitivity analyses for associations between single anthropometry measures and BC risk were presented in *Supplementary Table*5. Each 1 SD increment in BMI (5.1 kg/m^2^), WC (12.4 cm), WHR (0.1), and height (6.2 cm) was associated with 10% (95% CI: 7–14%), 12% (95% CI: 8–15%), 6% (95% CI: 3–9%), and 7% (95% CI: 5–9%) higher relative risk of BC, respectively, in the fully adjusted model.

### Biomarkers and Breast Cancer Risk

The multivariable-adjusted HRs for the associations between the biomarkers of interest and the risk of BC are presented in *Supplementary Table*6. Concentration levels of CRP, IGF-1, testosterone, gamma-glutamyltransferase, and urate were positively associated with BC risk, whereas HDL-cholesterol, SHBG, albumin, and apolipoprotein A were inversely related to BC risk. Triglycerides, alanine aminotransferase, cystatin C, and total bilirubin were not associated with BC risk.

### Four-Way Decomposition Mediation Analysis

As the association between PC and BC risk is a necessary condition for mediation analyses, these analyses were restricted to PC1 and PC2. The potential mediators were metabolic biomarkers which were associated with BC risk (*Supplementary Table*6). Furthermore, apart from total protein, cystatin C, and gamma-glutamyltransferase, all biomarkers of interest were either positively or inversely associated with PC1 or PC2 (*Supplementary Table*7). We considered causal effects for a change in PC1 and PC2 from the 25th to the 75th percentile, and each mediator fixed at its median level, after mutual adjustment for all other biomarkers.

### General Obesity Body Shape (PC1)

The results from the four-way decomposition of each potential mediator of the associations between PC1 and postmenopausal BC risk are shown in *Supplementary Table*8 (effect estimates) and Table 3 (attributable proportions). Overall, the CDE, the effect due to neither mediation nor interaction, showed strong positive associations between PC1 and BC risk across all 13 investigated biomarkers (i.e., mediators), with CDEs between 88.9% (95% CI: 82.2–95.6%), when holding testosterone levels fixed, to 101.9% (95% CI: 93.4–109.9%), when holding IGF-1 fixed at its median (Table 3* and Supplementary Fig. *12).

**Table 3:** Proportions attributable for the four-way decomposition of each mediator of the associations between a principal component 1 (PC1) of body shape (a general obese body shape) and postmenopausal breast cancer risk

	P_ CDE		P_ INTref		P_INTmed		P_PIE		OP_M	
Biomarkers	Proportion	P value	Proportion	P value	Proportion	P value	Proportion	P value	Proportion	P value
C-reactive protein (mg/L)	102.4%	< 0.001	-4.0%	0.060	-5.2%	0.089	6.7%	0.031	1.5%	0.660
HDL-cholesterol (mmol/L)	97.2%	< 0.001	2.2%	0.476	-1.1%	0.467	1.7%	0.592	0.6%	0.878
IGF-1 (nmol/L)	101.9%	< 0.001	10.3%	0.014	-8.2%	0.014	-4.1%	0.311	-12.3%	0.004
SHBG (nmol/L)	89.8%	< 0.001	5.2%	0.482	-5.4%	0.474	10.4%	0.112	5.0%	0.576
Testosterone (nmol/L)	88.9%	< 0.001	-0.4%	0.250	1.6%	0.442	9.9%	0.002	11.4%	< 0.001
Triglycerides (mmol/L)	102.1%	< 0.001	-1.5%	0.648	-0.3%	0.650	-0.3%	0.707	-0.6%	0.383
Albumin (g/L)	93.1%	< 0.001	0.9%	0.610	-1.6%	0.570	7.6%	0.036	6.0%	0.112
Glucose (mmol/L)	101.1%	< 0.001	-0.8%	0.479	-1.2%	0.497	1.0%	0.695	-0.3%	0.850
Alanine aminotransferase (U/L)	100.0%	< 0.001	0.0%	0.994	-0.2%	0.953	0.2%	0.954	0.0%	0.996
Apolipoprotein A (g/L)	98.2%	< 0.001	0.8%	0.514	0.6%	0.499	0.4%	0.844	1.0%	0.663
Gamma-glutamyltransferase (U/L)	101.7%	< 0.001	-1.8%	0.586	0.0%	0.746	0.1%	0.528	0.1%	0.612
Total bilirubin (umol/L)	99.5%	< 0.001	2.1%	0.721	-0.4%	0.718	-1.2%	0.190	-1.6%	0.166
Urate (umol/L)	102.9%	< 0.001	0.9%	0.661	-2.8%	0.662	-1.0%	0.886	-3.8%	0.535

P_CDE = proportion of controlled direct effect, P_INTref = proportion of reference interaction, P_INTmed = proportion of mediated interaction, P_PIE = proportion of pure indirect effect, OP_M = overall proportion mediated. Output of mediation analysis with causal effects estimated for a change in PC1 from the 25th to the 75th percentile. Controlled direct effects are computed fixing the mediators at their median levels

HDL cholesterol: high-density lipoprotein cholesterol, IGF-1: insulin-like growth factor, SHBG: sex hormone-binding globulin

Models were adjusted for age, center, healthy diet score, alcohol consumption frequency, smoking status, ethnicity, oral contraceptive, menopausal hormone treatment, physical activity, qualifications, Townsend deprivation index, and sedentary behavior, and mutually adjusted for biomarkers

There was a PIE (the effect only due to mediation, but not interaction) through IGF-1 and testosterone with mediated proportions equal to -4.1% (95% CI: -11.9 to 3.8%) and 10.4% (95% CI: 4 to 16%), respectively (Table 3* and Supplementary Fig. *12). The overall proportion mediated (sum of PIE and mediation that was activated because of an interaction of PC1 with each of the two biomarkers) was -12.3% (95% CI: -20.5% to -4.0%) and 11.4% (95% CI: 5.1 to 17.8%.) for IGF-1 and testosterone, respectively. There was no evidence for mediation by the other investigated biomarkers (Table 3* and Supplementary Table*8).

### Tall/Lean Body Shape (PC 2)

There was little variation in the proportion of CDE after fixing the mediators (i.e., 13 biomarkers) at their median values (Tables 4* and Supplementary Table*9). Minor proportions of the association between PC2 and BC were mediated by IGF-1 (PIE: 2.8%, 95% CI: 0.6 to 4.9%), and SHBG (PIE: -6.1%, 95% CI: -10.9% to -1.3%). There was no evidence of mediated interaction for IGF-1 (*P* = 0.242), while there was some indication of mediated interaction for SHBG (proportion mediated interaction: 3%, 95% CI: 0.7 to 6.0%) (Table 4* and Supplementary Fig. *13).

**Table 4:** Proportion attributable for the four-way decomposition of each mediator of the associations between principal component 2 (PC2) of body shape and postmenopausal breast cancer risk

	P_ CDE		P_ Int Ref		P_intmed		P_PIE		OP_M	
Biomarkers	Proportion	P value	Proportion	P value	Proportion	P value	Proportion	P value	Proportion	P value
C-reactive protein (mg/L)	98.3%	< 0.000	2.4%	0.709	-0.2%	0.707	-0.5%	0.242	-0.7%	0.133
HDL-cholesterol (mmol/L)	99.1%	< 0.000	2.1%	0.289	0.5%	0.285	-1.7%	0.174	-1.2%	0.335
IGF-1 (nmol/L)	101.6%	< 0.000	-3.0%	0.216	-1.3%	0.242	2.8%	0.012	1.4%	0.145
SHBG (nmol/L)	99.5%	< 0.000	3.5%	0.077	3.0%	0.045	-6.1%	0.012	-3.1%	0.115
Testosterone (nmol/L)	100.9%	< 0.000	-0.2%	0.942	-0.04%	0.856	-0.7%	0.186	-0.8%	0.172
Triglycerides (mmol/L)	103.5%	< 0.000	-5.1%	0.227	1.5%	0.251	0.2%	0.889	1.6%	0.323
Albumin (g/L)	97.8%	< 0.000	-0.3%	0.668	0.3%	0.738	2.2%	0.115	2.5%	0.059
Glucose (mmol/L)	101.4%	< 0.000	-1.4%	0.474	0.1%	0.548	-0.1%	0.664	0.1%	0.831
Alanine aminotransferase (U/L)	100.9%	< 0.000	-0.3%	0.892	0.1%	0.897	-0.7%	0.472	-0.6%	0.527
Apolipoprotein A (g/L)	96.5%	< 0.000	4.8%	0.097	-0.9%	0.091	-0.4%	0.668	-1.3%	0.262
Gamma-glutamyltransferase (U/L)	101.3%	< 0.000	-0.9%	0.837	0.1%	0.834	-0.5%	0.333	-0.4%	0.547
Total bilirubin (umol/L)	97.7%	< 0.000	0.6%	0.887	0.2%	0.885	1.5%	0.293	1.7%	0.171
Urate (umol/L)	100.5%	< 0.000	-0.4%	0.844	0.02%	0.890	-0.1%	0.627	-0.05%	0.731

P_CDE = proportion of controlled direct effect, P_INTref = proportion of reference interaction, P_INTmed = proportion of mediated interaction, P_PIE = proportion of pure indirect effect, OP_M = overall proportion mediated. Output of mediation analysis with causal effects estimated for a change in PC2 from the 25th to the 75th percentile. Controlled direct effects are computed fixing the mediators at their median levels

HDL cholesterol: high-density lipoprotein cholesterol, IGF-1: insulin-like growth factor, SHBG: sex hormone-binding globulin

Models were adjusted for age, center, healthy diet score, alcohol consumption frequency, smoking status, ethnicity, oral contraceptive, menopausal hormone treatment, physical activity, qualifications, Townsend deprivation index and sedentary behavior, and mutually adjusted for biomarkers.

### Sensitivity Analyses

Overall, sensitivity mediation analyses without mutual biomarkers adjustment showed no substantial differences as compared to mutual adjustment for PC1 (*Supplementary Tables*10 and 11). Regarding PC2, there was suggestive mediation of small proportions through CRP, HDL-cholesterol, SHBG, albumin, and urate, which however, are likely due to mediator-mediator confounding (*Supplementary Tables*12 and 13). Mediation analyses of the associations of BMI and WC with BC risk are shown in Supplementary Figs. 14 and 15. Results were generally in line with results for PC1. Minor proportions of the association between height and BC were mediated by IGF-1, SHBG, testosterone, and albumin (Supplementary Fig. 16).

## Discussion

Among 176,686 postmenopausal women enrolled in UK Biobank, a generally obese compared to a lean body shape (PC1), and a tall/lean compared to a short/centrally overweight body shape (PC2) were both associated with an increased risk of BC. The controlled direct effects (i.e., associations due to neither mediation nor interaction) were large, suggesting that most of the excess BC risk was due to other pathways than investigated here. Nevertheless, there was evidence that a pathway through testosterone mediated about 10% (i.e., pure indirect effect) of the positive association between body shape PC1 and the risk of BC. In contrast, IGF-1 and body shape PC1 were jointly associated with an increased risk of BC (i.e., reference interaction), and in addition, there was a mediated interaction, whereby body shape PC1 ‘causes’ IGF-1 and both were jointly associated with BC risk. This suggests that there are antagonistic associations between the joint association of PC1 and IGF-1 on BC risk (positive association), and the effect of PC1 on IGF-1 (inverse association) leading to a negative overall mediated proportion (-12%). For the association between body shape PC2 and BC risk, a small proportion (i.e., 2.8%) was mediated by IGF-1 (i.e., pure indirect effect). For SHBG, there was evidence for mediated interaction, but in opposite direction of the pure indirect effect, meaning that the overall proportion mediated was negligible. Sensitivity mediation analyses using BMI yielded comparable mediating effects to those of PC1.

In agreement with our study, a study conducted in EPIC reported an increased risk for BC in relation to both body shape PC1 (general adiposity) and body shape PC2 (tall; low WHR) [10]. These results are also congruent with previous studies investigating the association between obesity and risk of BC, but using single-trait anthropometric indicators such as BMI [1, 6, 13].

Using multi-trait body shapes has the advantage of removing redundant correlation between single anthropometric indicators (e.g., BMI is strongly correlated with WC), and thus potential confounding between them, which facilitates their use in statistical modelling. Furthermore, body shapes may capture phenotypic information that goes beyond single traits due to the way they combine. This is exemplified by a genome wide association study (GWAS) of our study group, where, for example, out of 678 genetic variants robustly associated with PC1 (p-value < 5 × 10-8), 21 variants were not previously linked to any of the six anthropometric traits [30]. Such additional genetic variation may translate into phenotypic differences that could also uncover novel mechanistic pathways explaining adiposity-cancer associations. However, the interpretation of the body shapes is less straightforward. To facilitate interpretation of the PCs of the body shapes, we provided the arithmetic means of each anthropometric trait among participants in the top and bottom 5% percentiles for the four body shape phenotypes, as well as the variation of these traits in the population (1 SD) (Supplementary Figs. 3–6). For PC1, for example, the difference in BMI between the top and bottom percentiles was 20 kg/m^2^, corresponding to 4 times the SD (5.1 kg/m^2^) in the study population. Similarly, there were significant differences in weight (4 times the SD), WC (3.9 times the SD), HC (3.9 times the SD), and WHR (2 times the SD). By contrast, the difference in height was 3 cm, corresponding to half of the SD in the study population. For PC2, the difference between the bottom and the top corresponded to 3.7 SD increment in height, 1.1 SD increment in weight and 1 SD increment in HC, while BMI, WC and WHR decreased by ≤ 1 SD.

Altered sex hormone metabolism is a main biological mechanism that could link excess adiposity with postmenopausal BC risk through increased aromatase enzyme activity in peripheral adipose tissue known as aromatization [31, 32]. Aromatization leads to increased levels of bioavailable sex hormones including testosterone, which may induce breast carcinogenesis [31]. Several epidemiological studies, including in UK Biobank, reported an increased BC risk associated with elevated blood levels of testosterone [15, 17]. Our study is in accordance with these findings and in addition, our mediation analysis supports the hypothesis that testosterone links general adiposity (i.e., body shape PC1) with postmenopausal BC risk.

There is strong evidence that higher IGF-I levels are associated with a greater risk of BC [33, 34]. Our findings for postmenopausal BC risk are congruent with this evidence (Supplementary Table 5). However, whether IGF-I links adiposity to BC is debated [31]. Levels of IGF-I increase only to a BMI of approximately 27 kg/m^2^, thereafter declining with increasing weight, and in individuals with overweight, who intentionally lose weight, IGF-1 levels tend to increase [31]. Our mediation analysis may shed some light on this uncertainty. While we did not observe a significant pure indirect effect through IGF-1, there was a reference interaction between PC1 and IGF-1, i.e., jointly increasing the risk of BC (‘joint effect’) beyond their individual risk associations. In addition, there was a mediated interaction between PC1 and IGF-1 leading to a negative overall proportion mediated of 12% (Table 3).

In contrast, we estimated a small but clearcut mediation of 2.8% (i.e., proportion of pure indirect effect) through IGF-1 for the association between PC2 (tall, low WHR) and BC risk. IGF-1 signaling is well known to induce expression of several oncogenes and high concentrations of IGF-1 are associated with increased risk of BC [35]. Because several genetic variants related to the IGF signaling pathway are related to height, height might be a crude anthropometric marker of early-life IGF-1 exposure [35]. Our results confirm that a proportion, albeit small, of the height-BC relationship is mediated through IGF-1.

We identified a second molecular pathway linking PC2 with BC risk, which was through SHBG. Since the height component of this body shape was positively associated with SHBG, and in turn SHBG was inversely associated with the risk of BC, the mediated proportion was negative (‘pure indirect effect’: -6%). This is in line with evidence that higher levels of SHBG lead to lower levels of bioavailable testosterone and estrogens, and thus a lower BC risk via this pathway [15, 16]. A meta-analysis of 26 prospective studies showed that high SHBG levels were significantly associated with decreased risk of BC in postmenopausal women, the pooled RR for BC comparing the highest vs. lowest categories of SHBG was 0.64 (95% CI: 0.57–0.72) [36]. In a recent study from UK Biobank, SHBG was inversely associated with BC risk in postmenopausal women [16]. The mediating effect of SHBG in the association between adiposity and BC development has not been investigated in other studies yet. However, SHBG has been reported to mediate a small proportion of the relationship between BMI and endometrial cancer risk (7%) [37], and the alcohol-BC association (13%) [38].

Our findings suggest differential pathways linking these body shapes with BC risk. Indeed, PC1 may have similar pathways as obesity measured by BMI, while PC2 may have different biological/metabolic pathways involved in carcinogenesis of BC, more comparable to height.

There have been many advances in mediation analysis methodology over the years, with various methods of mediation analysis reported in the literature. One of the novelties of this study is that we considered both the mediation and interaction pathways simultaneously using one of the most recent approaches to causal mediation analysis: “the four-way decomposition”. This approach allows us to estimate both the pure indirect and interaction effect, as compared to previous studies using conventional approaches for mediation analysis which may have missed mediated interaction effects. The present study is also the first to investigate the potential mediator role of several biomarkers of metabolic health in the association between body shape phenotypes and BC development, as compared to previous studies investigating classical anthropometric parameters (mainly BMI or WHR). Finally, we accounted for confounding by other biomarkers. Limitations of this study included the lack of data on hormone receptor status since the relationship between obesity and BC risk may differ according to estrogen or progesterone receptor. We lacked sufficient individuals with oestradiol measurements to investigate this sex-hormone as potential mediator which has been both related to obesity and BC development. Also, we could not rule out the possibility of unmeasured confounding. Lack of representativeness of the study sample for the general population is acknowledged.

In the context of two or more mediators explaining an exposure-outcome association, as for example in the current study, where levels of testosterone and IGF-1 explained each about 10% of the body shape PC1-BC association, it is conceivable that mediators also interact with each other. This requires that the 4-way decomposition is extended to accommodate such a potential mediator-mediator interaction [39] which, however, was beyond the scope of the current study. Alternatively, future studies could consider dimension reduction approaches, such as PCA, to derive one or more patterns from multiple correlated biomarkers, and subsequently use these in a mediation framework.

## Conclusion

In summary, although the direct effects of body shape phenotypes on BC risk are high, our findings are consistent with a possible mediated effect by testosterone and IGF-1 for the body shape PC1 and BC development association, while IGF-1 and SHBG may have a role in the association between body shape PC2 and BC development. As the mediating biomarkers may interact with each other, future studies should also consider a decomposition of the total effect in the presence of multiple mediators, particularly by family of biomarkers.

### Electronic Supplementary Material

Below is the link to the electronic supplementary material.

Supplementary Material 1

## Data Availability

The UK Biobank resource is available to bona fide researchers for health-related research in the public interest. All researchers who wish to access the research resource must register with UK Biobank by completing the registration form in the Access Management System (AMS- https://ams.ukbiobank.ac.uk/ams/). UK Biobank data can be provided by UK Biobank Limited pending scientific review and a completed material transfer agreement.
