# Supplementary material for: Multi-Trait Body Shape Phenotypes and Breast Cancer Risk in Postmenopausal Women: A Causal Mediation Analysis in the UK Biobank Cohort
Source: J Epidemiol Glob Health. 2024 Apr 10;14(2):420–32. doi: 10.1007/s44197-024-00226-4 (PMC11176278; doi:10.1007/s44197-024-00226-4)
Supplement: Supplementary file 1 — Supplementary Material 1 [file 44197_2024_226_MOESM1_ESM.docx]

**Supplementary material**

**Multi-trait body shape phenotypes and breast cancer risk in postmenopausal women: a causal mediation analysis in the UK Biobank cohort**

Amina Amadou ^1 2^, Heinz Freisling ^3^, Anja M. Sedlmeier ^4^, Patricia Bohmann^4^, Emma Fontvieille ^3^, Andrea Weber ^4^, Julian Konzok ^4^, Michael J Stein ^4^, Laia Peruchet-Noray ^3^, Anna Jansana^3^, Hwayoung Noh ^1 2^, Mathilde His ^1 2^, Quan Gan ^3^, Hansjörg Baurecht ^4^, Béatrice Fervers ^1 2^

Affiliations :

^1^ Department of Prevention Cancer Environment, Centre Léon Bérard, Lyon, France

^2^ Inserm U1296 Radiations : Défense, Santé, Environnement, Lyon, France

^3^ International Agency for Research on Cancer (IARC), Nutrition and Metabolism Branch, Lyon, France

^4^ Department of Epidemiology and Preventive Medicine, University of Regensburg, Regensburg, Germany

**Corresponding author**

Amina Amadou

Département Prévention Cancer Environnement, Centre Léon Bérard

Inserm U1296 Radiations : Défense, Santé, Environnement

28 rue Laënnec, 69373 Lyon Cedex 08, France.

Phone : +33(0)426556804

E-mail : [amina.amadou@lyon.unicancer.fr](mailto:amina.amadou@lyon.unicancer.fr)

Premenopausal women

n = 60,910

Women with missing data on menopausal status, and were aged < 55 years n = 8,760

UK Biobank study baseline

as of August 02, 2022

n = 502,384

Overall participants

n = 465,419

Participants with prevalent cancer (diagnosis before baseline assessment)

n = 36,964

Women without prevalent cancer

n=247,798

Overall women participants n=246,356

Dataset for analysis

n = 176,686

Breast cancer cases/ non-cases = 6,396/170,290

Participants without sex information

n = 1

Men participants

n = 217,621

Women with missing values, and with aberrant data on exposures (BMI < 16 kg/m^2^, height < 130 cm, WC < 40 cm, WC >160 cm, HC < 60 cm)

n= 1,442

**Supplementary Figure 1: Flowchart of the selection of the study participants.**

BMI: body mass index, WC: waist circumference, HC: hip circumference.

The sample size of “176,686 participants” is the initial population used for the crude analysis models that were stratified only by age at recruitment in 5-year categories, and study center, without any missing values. The multivariable-adjusted analyses were then conducted in the complete-case dataset, excluding all women with a missing value (n = 47,319) for any of the adjusted covariates, resulting in a final sample size of 129,367 participants.


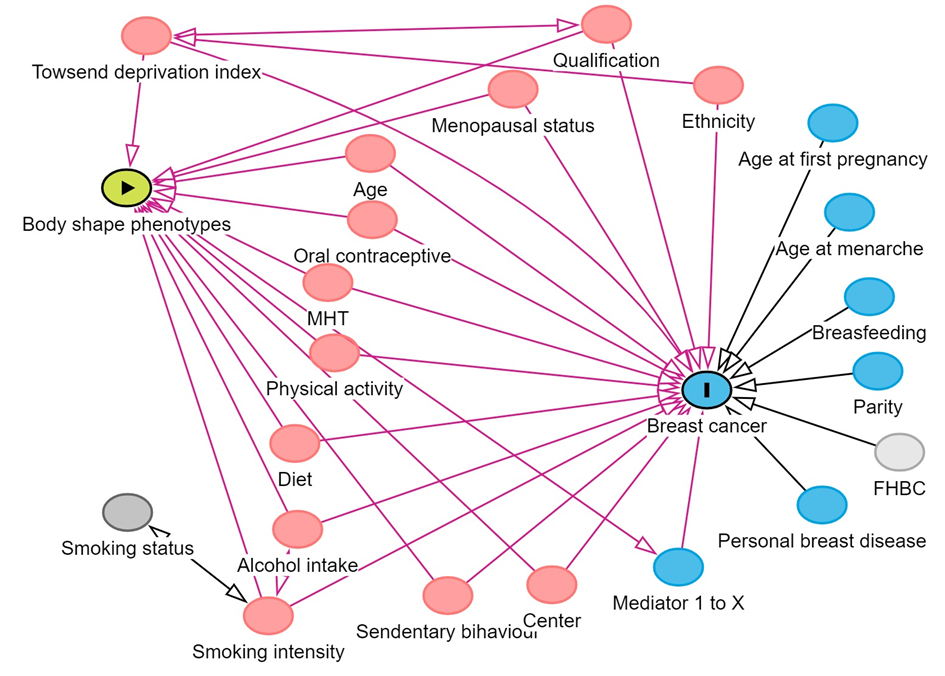


**Supplementary Figure 2: Directed Acyclic Graph showing the relationship between potential confounders in the association between body shape phenotypes and breast cancer risk.**

MHT: use of menopausal hormone treatment, FHBC: family history of breast cancer.


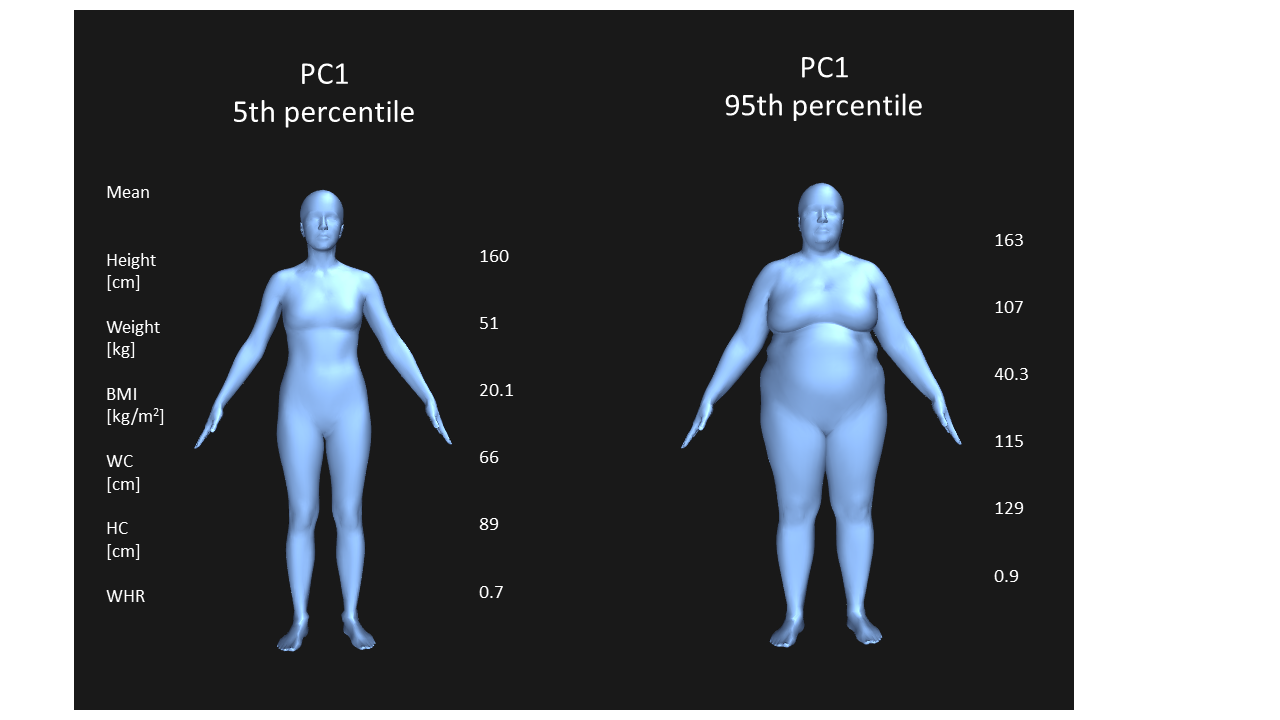


**Supplementary Figure 3: Visualization of body shapes for the first principal component (PC1).**

For the plot, mean values of anthropometric measures of the top (p95) and bottom (P5) of the UK Biobank study population were calculated for PC1, using <https://bodyvisualizer.com/>

For better interpretation, we provide the population standard deviation of each of the six anthropometric measures: height [cm] 6.2; weight [kg] 13.8; BMI [kg/m^2^] 5.1; WC [cm] 12.4; HC [cm] 10.3; WHR 0.1.


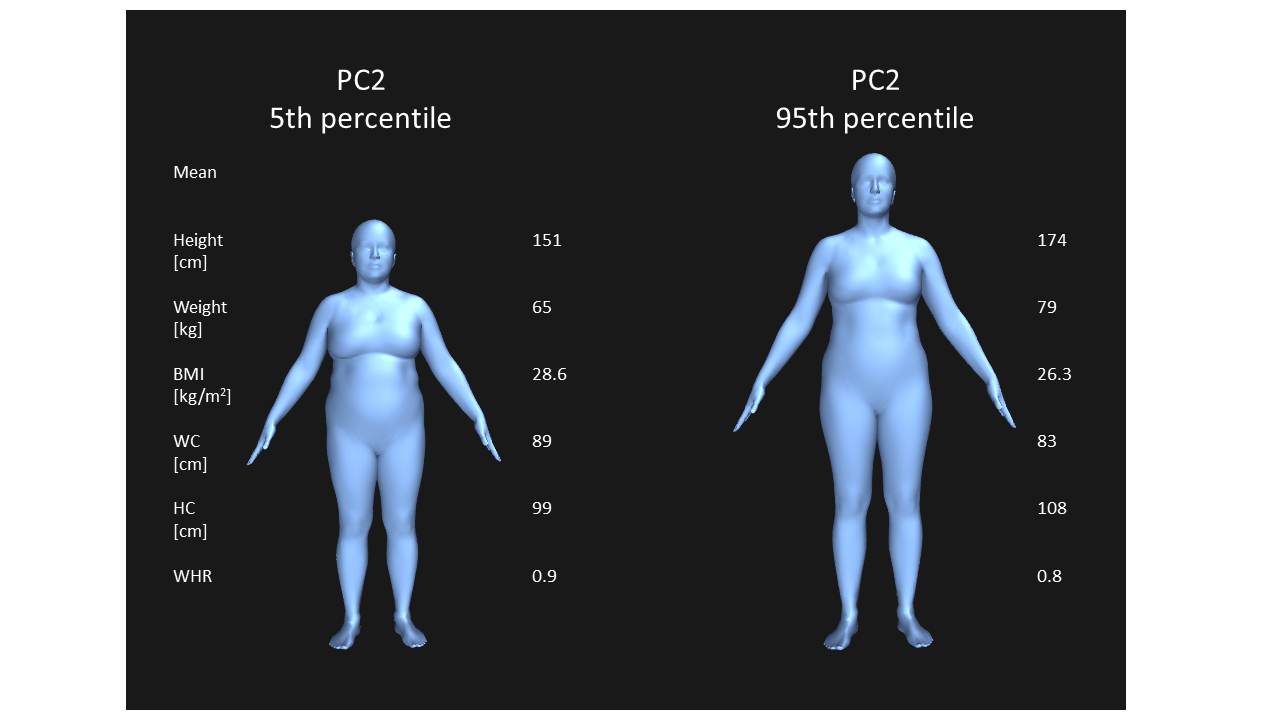
**Supplementary Figure 4: Visualization of body shapes for the second** **principal component (PC2).**

For the plot, mean values of anthropometric measures of the top (p95) and bottom (P5) of the UK Biobank study population were calculated for PC2, using <https://bodyvisualizer.com/>.

For better interpretation, we provide the population standard deviation of each of the six anthropometric measures: height [cm] 6.2; weight [kg] 13.8; BMI [kg/m^2^] 5.1; WC [cm] 12.4; HC [cm] 10.3; WHR 0.1.


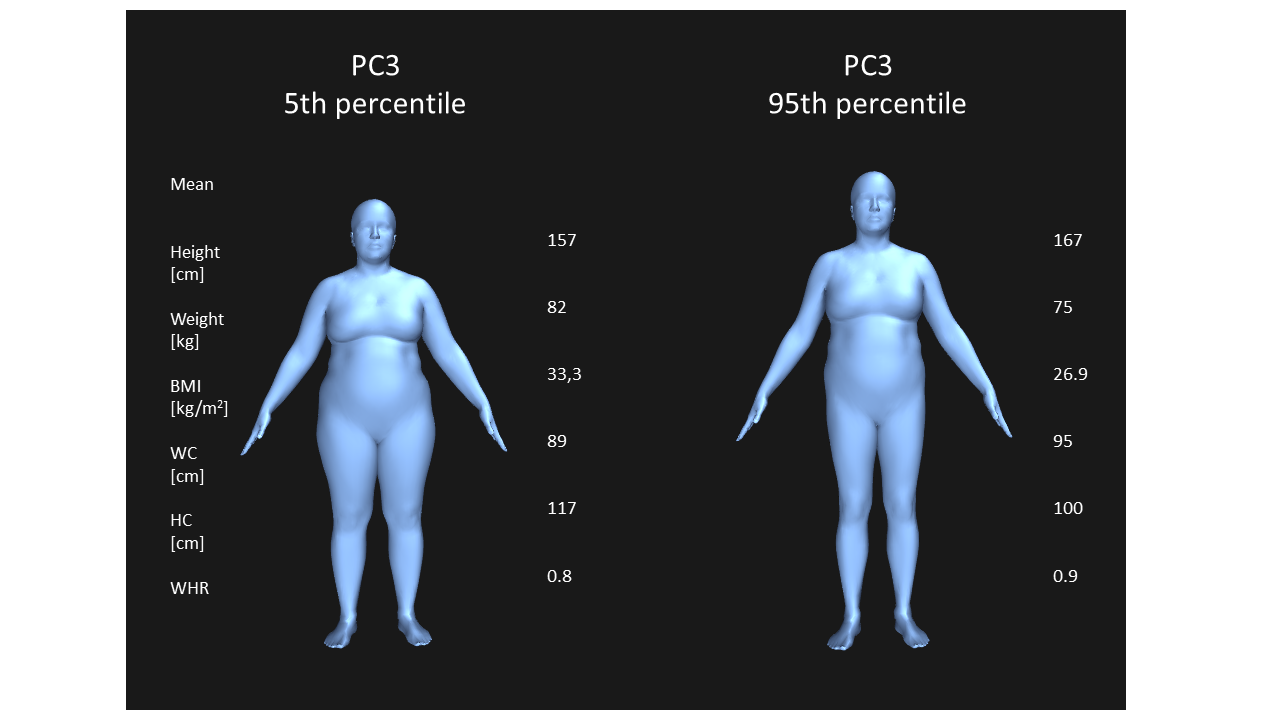
**Supplementary Figure 5: Visualization of body shapes for the third principal component (PC3).**

For the plot, mean values of anthropometric measures of the top (p95) and bottom (P5) of the UK Biobank study population were calculated for PC3, using <https://bodyvisualizer.com/>.

For better interpretation, we provide the population standard deviation of each of the six anthropometric measures: height [cm] 6.2; weight [kg] 13.8; BMI [kg/m^2^] 5.1; WC [cm] 12.4; HC [cm] 10.3; WHR 0.1.


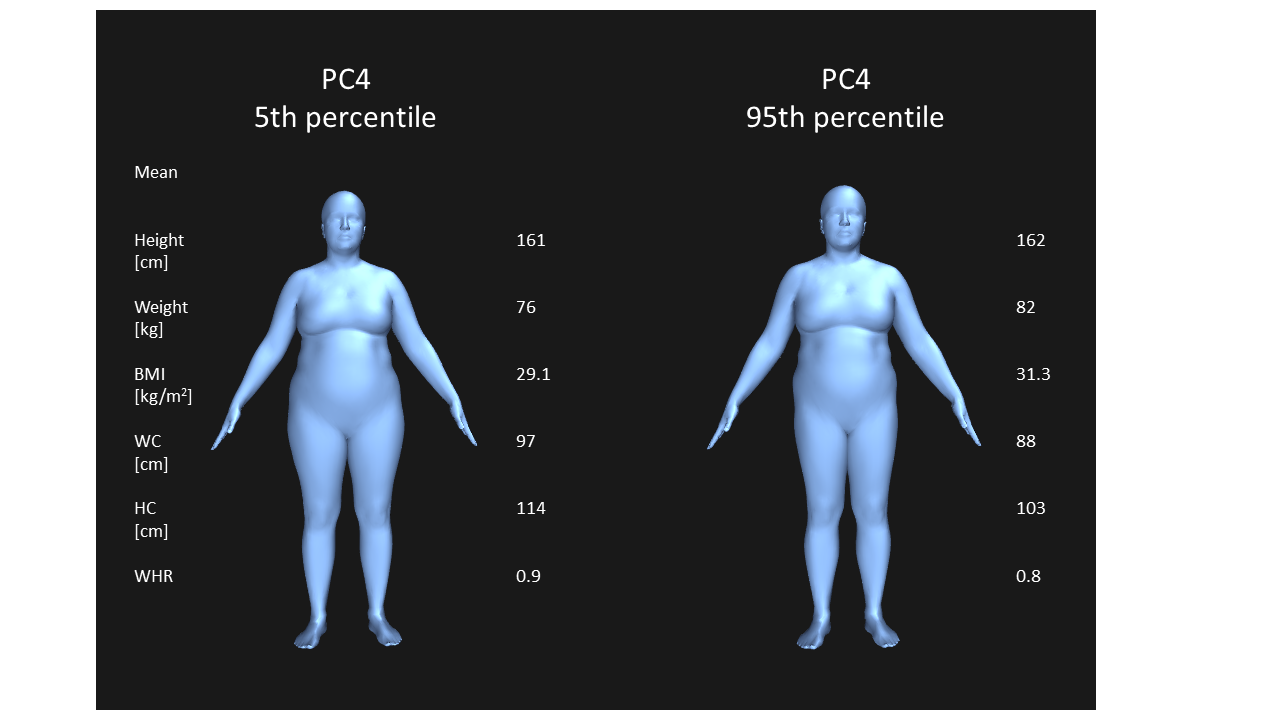
**Supplementary Figure 6: Visualization of body shapes for the fourth** **principal component (PC4).**

For the plot, mean values of anthropometric measures of the top (p95) and bottom (P5) of the UK Biobank study population were calculated for PC4, using <https://bodyvisualizer.com/>.

For better interpretation, we provide the population standard deviation of each of the six anthropometric measures: height [cm] 6.2; weight [kg] 13.8; BMI [kg/m^2^] 5.1; WC [cm] 12.4; HC [cm] 10.3; WHR 0.1.


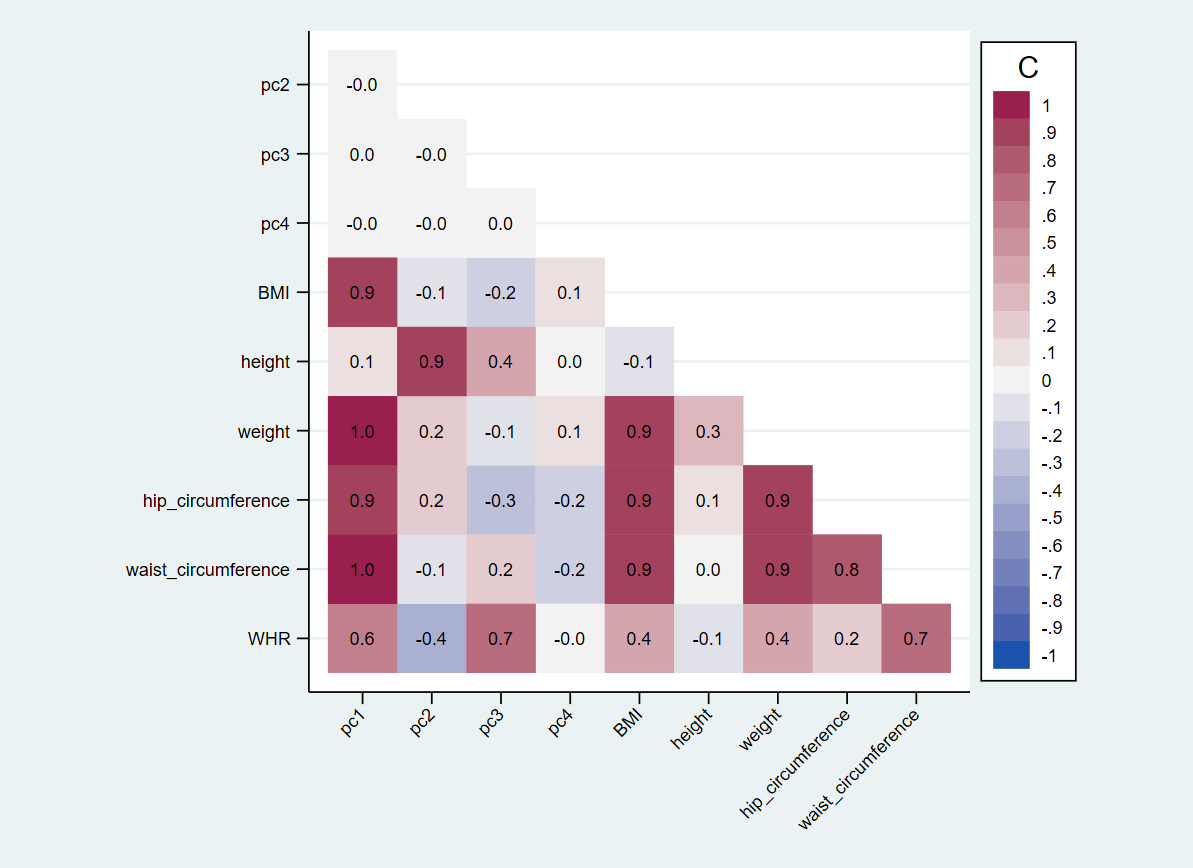


**Supplementary Figure 7: Pearson's correlation matrix between the six anthropometric measures and PCs.**

C: coefficients of correlation, PC: principal component, BMI: body mass index, WHR: waist-to-hip ratio,

**Supplementary Table 1**: Baseline characteristics of postmenopausal women by breast cancer status from the UK Biobank cohort study

| **Characteristics** | **Cases**  **(n = 6,396)** | **Non cases**  **(n = 170,290)** |
| --- | --- | --- |
| Age at recruitment (years), mean (SD) | 61.0 (5.3) | 60.4 (5.7) |
| Age at menarche (years), mean (SD) | 12.9 (1.6) | 12.9 (1.6) |
| Townsend deprivation index, mean (SD) | -1.5 (2.9) | -1.4 (2.9) |
| Sedentary behaviour, mean (SD) | 4.51 (2.1) | 4.47 (2.1) |
| BMI (kg/m²), mean (SD) | 27.7 (5.1) | 27.3 (5.1) |
| Weight (kg), mean (SD) | 73.2 (13.7) | 71.4 (13.8) |
| Height(m), mean (SD) | 1.63 (0.1) | 1.62 (0.1) |
| WHR, mean (SD) | 0.83 (0.1) | 0.82 (0.1) |
| Waist circumference (cm), mean (SD) | 86.8 (12.3) | 85.4 (12.4) |
| Hip circumference (cm), mean (SD) | 104.6 (10) | 103.6 (10.3) |
| Physical activity, n (%) |  |  |
| Low | 907 (18.9) | 23,386 (18.2) |
| Moderate | 2,169 (45.2) | 55,589 (43.1) |
| High | 1,724 (35.9) | 49,910 (38.7) |
| Alcohol drinking, n (%) |  |  |
| Daily or almost daily | 1,206 (18.9) | 28,747 (16.9) |
| 3 to 4 times a week | 1,325 (20.7) | 33,820 (19.9) |
| 1 to 2 times a week | 1,544 (24.2) | 42,343 (24.9) |
| 1 to 3 times a month | 775 (12.1) | 21,254 (12.5) |
| Special occasions only | 941 (14.7) | 26,598 (15.7) |
| Never | 596 (9.3) | 17,233 (10.1) |
| Smoking status, n (%) |  |  |
| Never | 3,601 (56.5) | 99,292 (58.6) |
| Previous | 2,207 (34.7) | 56,246 (33.2) |
| Current | 560 (8.8) | 13,918 (8.2) |
| Qualification, n (%) |  |  |
| None of the above | 1,333 (21.3) | 35,931 (21.5) |
| College or University degree | 1,803 (28.8) | 46,864 (28.1) |
| A levels/AS levels or equivalent | 709 (11.3) | 18,075 (10.8) |
| O levels/GCSEs or equivalent | 1,506 (24.0) | 39,299 (23.5) |
| CSEs or equivalent | 248 (3.9) | 7,385 (4.4) |
| NVQ or HND or HNC or equivalent | 275 (4.4) | 8,073 (4.8) |
| Other professional | 389 (6.2) | 11,389 (6.8) |
| Ever use of oral contraceptive, n (%) |  |  |
| No | 1,406 (22.1) | 36,877 (21.7) |
| Yes | 4,966 (77.9) | 132,772 (78.3) |
| Ever use of menopausal hormone therapy, n (%) | |  |
| No | 2,969 (46.6) | 83,903 (49.5) |
| Yes | 3,397 (53.4) | 85,694 (50.5) |
| Healthy diet score, n (%) |  |  |
| 0 (unhealthy) | 4 (0.1) | 134 (0.1) |
| 1 | 54 (0.8) | 1,328 (0.8) |
| 2 | 301 (4.7) | 7,611 (4.5) |
| 3 | 1,387 (21.7) | 36,621 (21.5) |
| 4 | 2,992 (46.8) | 79,577 (46.7) |
| 5 | 1,472 (23.0) | 39,495 (23.2) |
| 6 (healthy) | 187 (2.9) | 5,524 (3.2) |
| Screening mammogram, n (%) |  |  |
| No | 209 (3.3) | 9,255 (5.4) |
| Yes | 6,177 (96.7) | 160,737 (94.6) |
| Ethnicity, n (%) |  |  |
| White | 6,158 (96.6) | 162,088 (95.5) |
| Mixed | 24 (0.4) | 821 (0.5) |
| Asian | 89 (1.4) | 2,538 (1.5) |
| Black | 56 (0.9) | 2,331 (1.4) |
| Chinese | 10 (0.1) | 499 (0.3) |
| Other ethnic group | 37 (0.6) | 1,385 (0.8) |

Mean (Standard deviation) and Counts (Percentages) are presented for continuous and categorical variables respectively.

BMI: body mass index, WHR: waist-to-hip ratio, healthy diet score (from unhealthy to healthy) was calculated based on consumption of these commonly food groups (fruits, vegetables, fish, processed meats, unprocessed red meats, whole grains, and refined grains) [28], Sedentary behaviour = sum of time spent watching television, [time spent using computer](https://biobank.ndph.ox.ac.uk/ukb/field.cgi?id=1080) and time spent driving, Qualification : A: advanced, AS: advanced subsidiary, O: ordinary, GCSE: general certificate of secondary education, CSE: certificate of secondary education, NVQ: national vocational qualification, HND: higher national diploma, HNC: higher national certificate

**Supplementary Table 2**: Distribution of the main biomarkers of postmenopausal women from the UK Biobank cohort study

|  | **Cases** | | | **Non cases** | |
| --- | --- | --- | --- | --- | --- |
| **Biomarkers** | **Median** | | **P25-P75** | **Median** | **P25-P75** |
| Cholesterol (mmol/L) | | 6.00 | 5.26 - 6.76 | 6.01 | 5.27 -6.76 |
| C-reactive protein (mg/L) | | 1.68 | 0.83 - 3.44 | 1.48 | 0.72 -3.11 |
| Glycated haemoglobin (HbA1c) (mmol/mol) | | 35.8 | 33.5 - 38.3 | 35.9 | 33.6 -38.3 |
| HDL-cholesterol (mmol/L) | | 1.56 | 1.32 - 1.84 | 1.57 | 1.34 -1.84 |
| IGF-1 (nmol/L) | | 20.2 | 16.4 - 23.8 | 19.9 | 16.3 -23.5 |
| SHBG (nmol/L) | | 52.2 | 37.0 - 70.9 | 54.8 | 39.0 -74.7 |
| Testosterone (nmol/L) | | 1.02 | 0.74 - 1.40 | 0.97 | 0.69 -1.32 |
| Total protein (g/L) | | 72.1 | 69.6 - 74.8 | 72.3 | 69.7 -75.0 |
| Triglycerides (mmol/L) | | 1.46 | 1.06 - 2.02 | 1.42 | 1.04 -2.0 |
| Albumin (g/L) | | 45.0 | 43.2 - 46.6 | 45.0 | 43.3 - 46.7 |
| Glucose (mmol/L) | | 4.99 | 4.66 - 5.35 | 4.96 | 4.64 - 5.33 |
| Alanine aminotransferase (U/L) | | 18.6 | 14.9 - 24.6 | 18.3 | 14.6 -23.9 |
| Apolipoprotein A (g/L) | | 1.63 | 1.45 - 1.81 | 1.63 | 1.47 - 1.81 |
| Apolipoprotein B (g/L) | | 1.05 | 0.90 - 1.21 | 1.05 | 0.90 - 1.21 |
| Cystatin C (mg/L) | | 0.89 | 0.81 - 0.98 | 0.88 | 0.80 - 0.98 |
| Gamma-glutamyltransferase (U/L) | | 23.6 | 17.6 - 35.9 | 22.6 | 17 - 33.8 |
| Total bilirubin (umol/L) | | 7.27 | 5.93 - 9.16 | 7.23 | 5.92 -9.1 |
| Urate (umol/L) | | 275.5 | 235.4 - 321.4 | 270.9 | 231.3 - 316.4 |

HDL cholesterol: high-density lipoprotein cholesterol, IGF1: insulin-like growth factor, SHBG: sex hormone-binding globulin.

| **Supplementary Table 3**: Loadings and explained variance of the principal components for women | | | | | | |
| --- | --- | --- | --- | --- | --- | --- |
|  | PC1 | PC2 | PC3 | PC4 | PC5 | PC6 |
| Height | 0.043 | 0.851 | 0.449 | 0.022 | 0.269 | -0.012 |
| Weight | 0.481 | 0.226 | -0.086 | 0.489 | -0.686 | 0.033 |
| BMI | 0.479 | -0.106 | -0.272 | 0.479 | 0.675 | -0.027 |
| Hip | 0.457 | 0.182 | -0.353 | -0.609 | 0.015 | 0.511 |
| Waist | 0.489 | -0.111 | 0.184 | -0.399 | -0.037 | -0.745 |
| WHR | 0.300 | -0.411 | 0.747 | 0.028 | 0.021 | 0.427 |
| Explained variance (%) | 65.71 | 19.10 | 13.50 | 1.61 | 0.04 | 0.04 |

PC: principal component, BMI: body mass index, WHR: waist-to-hip ratio.


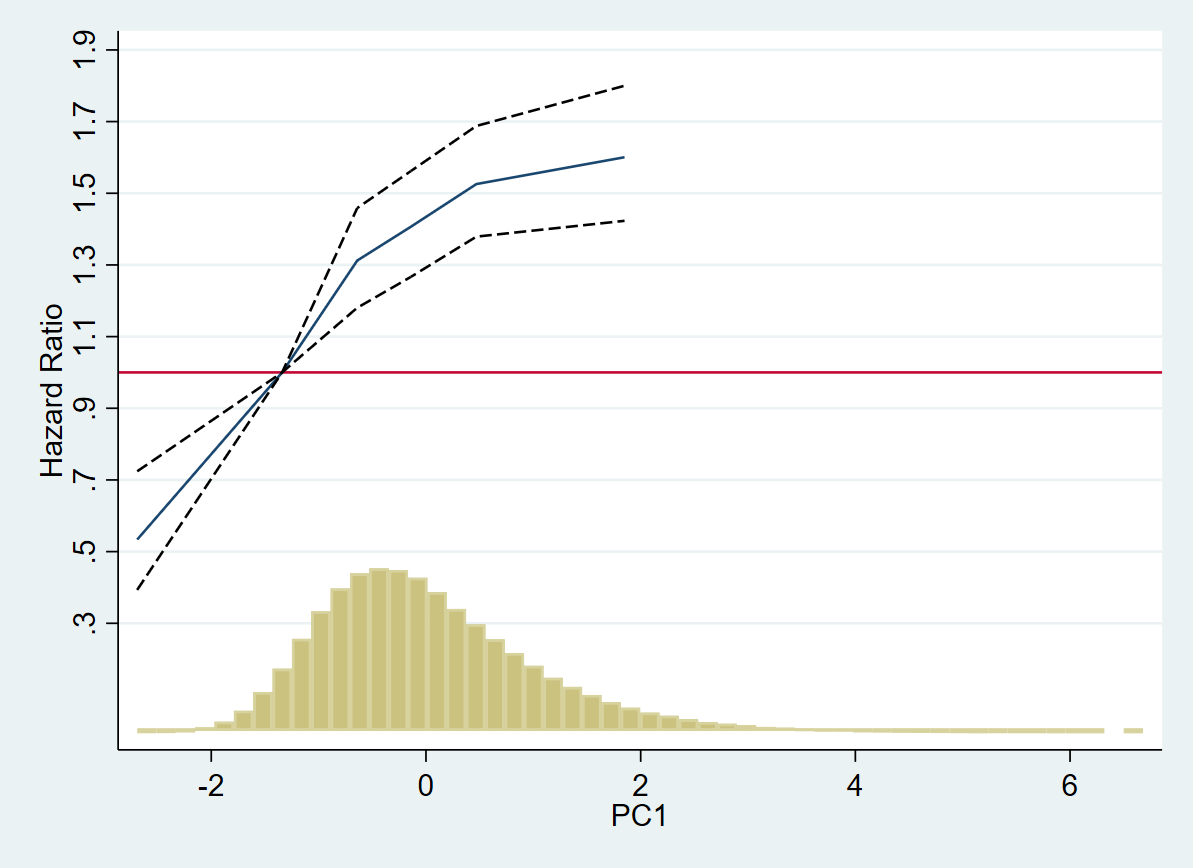


**Supplementary Figure 8: Cubic spline modelling of the relationship between PC1 and postmenopausal breast cancer risk.** Multivariable adjusted hazard ratios (continuous line) and 95% confidence intervals (dotted line) obtained using five-knot restricted cubic splines with the P5th value used as reference. Models were stratified by age at recruitment in 5-year categories, study center, and adjusted for alcohol intake, smoking status, ethnicity, oral contraceptive, menopausal hormone treatment, physical activity, qualifications, Townsend deprivation index and sedentary behavior.


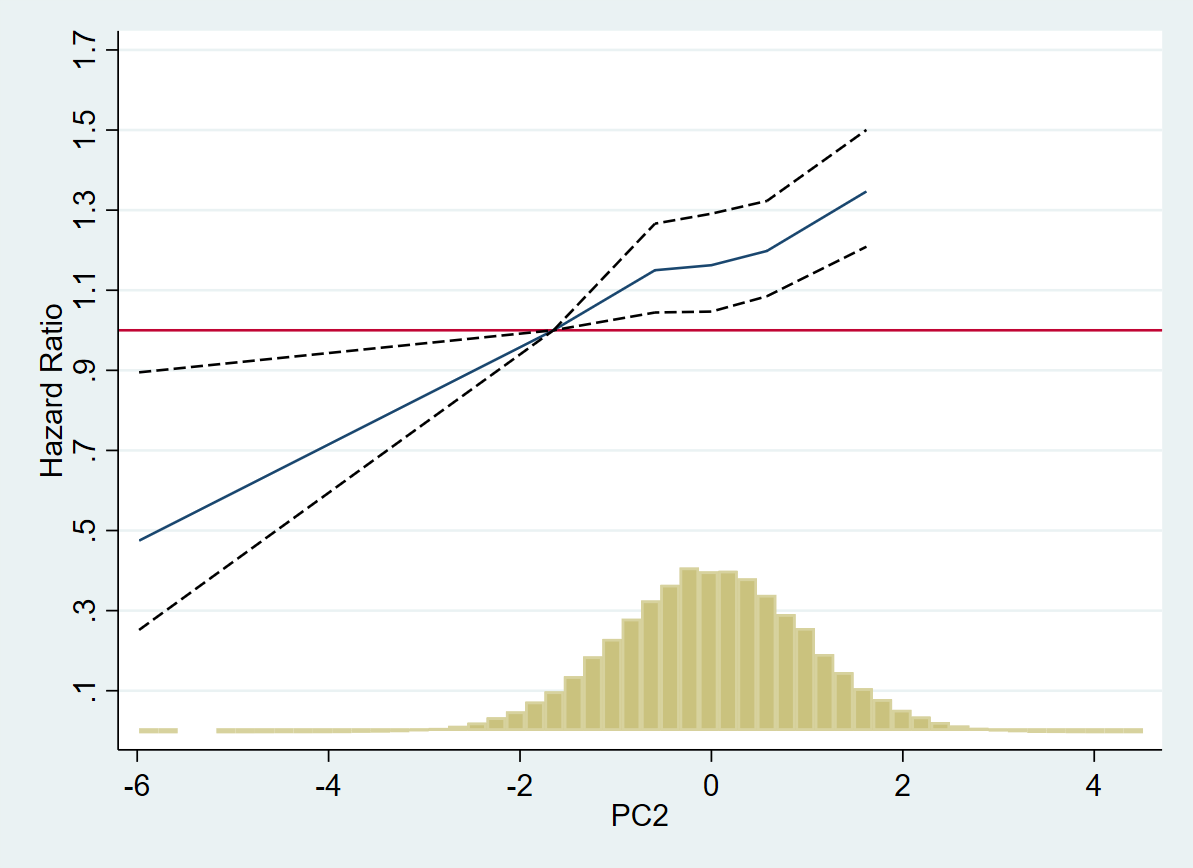


**Supplementary Figure 9: Cubic spline modelling of the relationship between PC2 and postmenopausal breast cancer risk.** Multivariable adjusted hazard ratios (continuous line) and 95% confidence intervals (dotted line) obtained using five-knot restricted cubic splines with the P5th value used as reference. Models were stratified by age at recruitment in 5-year categories, study center, and adjusted for alcohol intake, smoking status, ethnicity, oral contraceptive, menopausal hormone treatment, physical activity, qualifications, Townsend deprivation index and sedentary behavior.


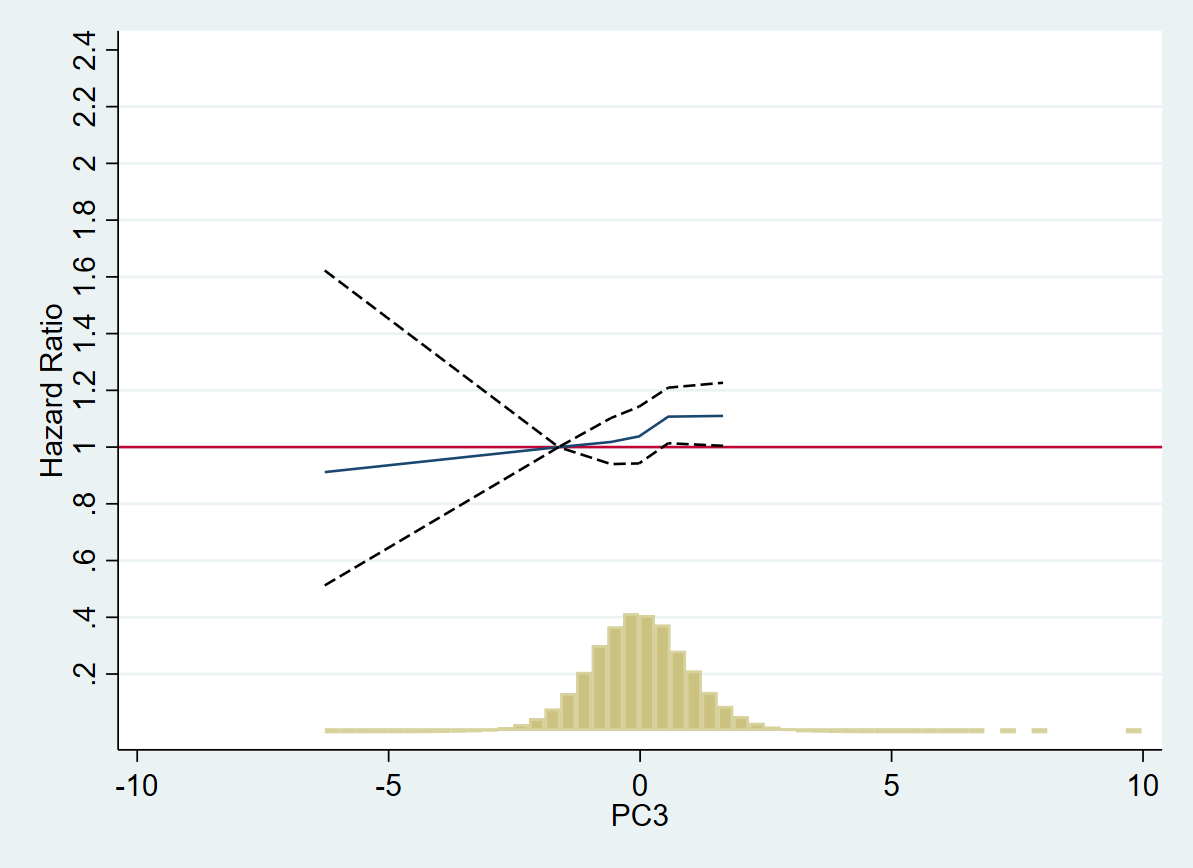


**Supplementary Figure 10 Cubic spline modelling of the relationship between PC3 and postmenopausal breast cancer risk.** Multivariable adjusted hazard ratios (continuous line) and 95% confidence intervals (dotted line) obtained using five-knot restricted cubic splines with the P5th value used as reference. Models were stratified by age at recruitment in 5-year categories, study center, and adjusted for alcohol intake, smoking status, ethnicity, oral contraceptive, menopausal hormone treatment, physical activity, qualifications, Townsend deprivation index and sedentary behavior.


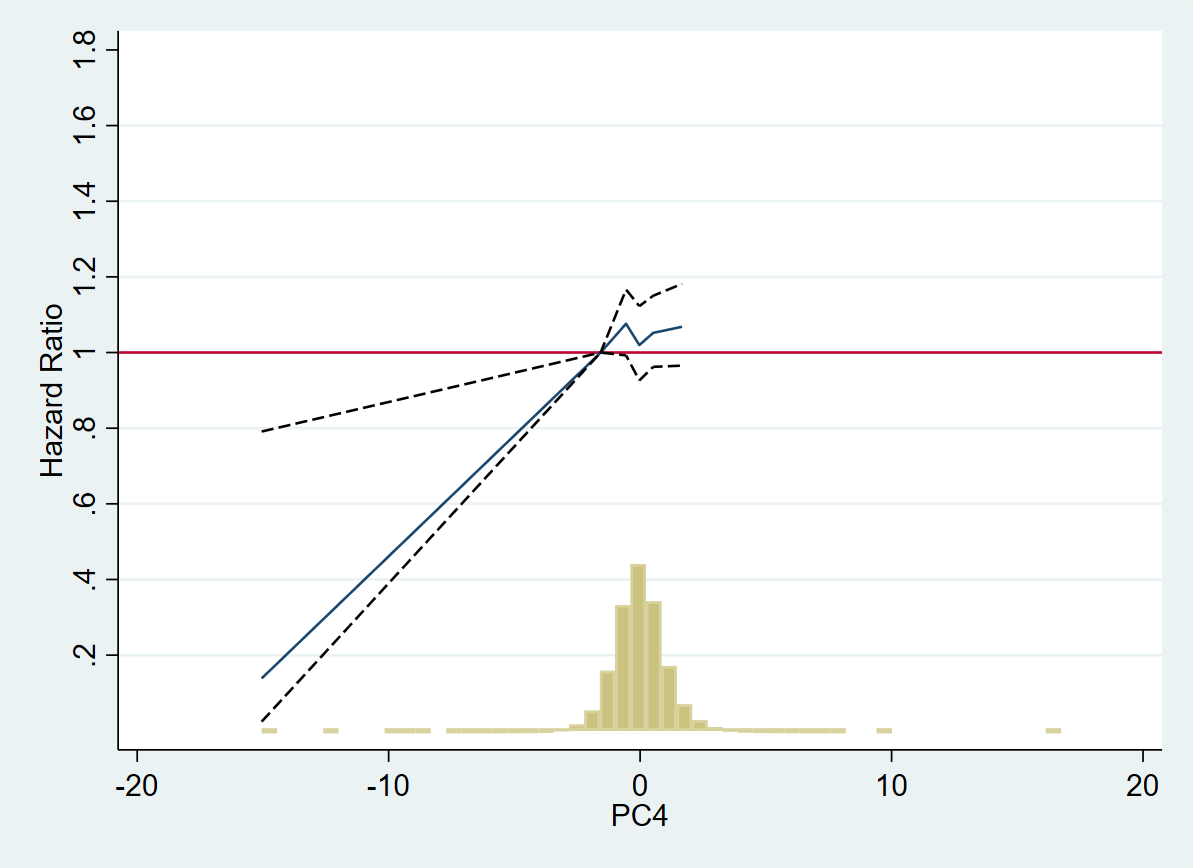

**Supplementary Figure 11: Cubic spline modelling of the relationship between PC4 and postmenopausal breast cancer risk.** Multivariable adjusted hazard ratios (continuous line) and 95% confidence intervals (dotted line) obtained using five-knot restricted cubic splines with the P5th value used as reference. Models were stratified by age at recruitment in 5-year categories, study center, and adjusted for alcohol intake, smoking status, ethnicity, oral contraceptive, menopausal hormone treatment, physical activity, qualifications, Townsend deprivation index and sedentary behavior.

**Supplementary Table 4:** Associations between principal components and postmenopausal breast cancer risk (per 1 SD increment)

|  | | **PC1** | | | **PC2** | | **PC3** | | **PC4** | |
| --- | --- | --- | --- | --- | --- | --- | --- | --- | --- | --- |
|  | Cases/non cases | | HR (95% CI) | Cases/non cases | | HR (95% CI) | Cases/non cases | HR (95% CI) | Cases/non cases | HR (95% CI) |
| **Sensitivity analyses** | | |  |  | |  |  |  |  |  |
| 1 | 3,850 /123,406 | | 1.13 (1.09-1.17) | 3,850 /123,406 | | 1.08 (1.05-1.12) | 3,850 /123,406 | 1.03 (1.00-1.07) | 3,850 /123,406 | 1.02 (0.98-1.05) |
| 2 | 6,396/170,290 | | 1.13 (1.11-1.16) | 6,396/170,290 | | 1.10 (1.07-1.12) | 6,396/170,290 | 1.05 (1.02-1.08) | 6,396/170,290 | 1.03 (1.00-1.06) |
| 3 | 3,912/108,842 | | 1.14 (1.10-1.18) | 3,912/108,842 | | 1.08 (1.04-1.11) | 3,912/108,842 | 1.04 (1.00-1.07) | 3,912/108,842 | 1.00 (0.97-1.03) |

Hazard ratios per 1 standard deviation (SD) increment in each PC from Cox proportional hazards regression using age as the underlying time metric. Stratified by age in 5-year categories, center and diet, and adjusted for age alcohol frequency, smoking status, ethnicity, oral contraceptive, menopausal hormone treatment, physical activity, qualifications, townsend deprivation index and sedentary behavior. All four principal components were mutually adjusted.

1: Excluding participants with the first 2 years of follow-up, n = 127,256

2: Multiple imputation, n =176,686

3: Exclusively on postmenopausal women, who answered having their periods stopped at least 1 year, n = 108,754

**Supplementary Table 5:** Associations between single anthropometric measures and postmenopausal breast cancer risk

| Continuous (for an increment of 1 SD) | Cases/non cases | Multivariable HR (95% CI) |
| --- | --- | --- |
| BMI | 4,671/125,138 | 1.10 (1.07-1.14) |
| WC | 4,671/125,138 | 1.12 (1.08-1.15) |
| WHR | 4,671/125,138 | 1.06 (1.03-1.09) |
| Height | 4,671/125,138 | 1.07 (1.04-1.09) |

SD: standard deviation, BMI: body mass index, WC: waist circumference, WHR: waist-to-hip ratio, WHTR: waist-to-height ratio

Hazard ratios from Cox proportional hazards regression using age as the underlying time metric. Multivariable models were stratified by age at recruitment in 5-year categories, study center, and adjusted for alcohol intake, smoking status, ethnicity, oral contraceptive, menopausal hormone treatment, physical activity, qualifications, Townsend deprivation index and sedentary behavior.

The HR (95% CI) corresponds to an increment of 1 SD: BMI=5.1 kg/m^2^, WC=12.4 cm, WHR=0.1, WHTR= 0.08, Height=6.2cm

**Supplementary Table 6**: Hazard ratios and 95% CI for the association of baseline levels of metabolic and biological markers with risk of incident post-menopausal breast cancer

| **Biomarkers** | **Cases** | **Non cases** | **HR (95% CI)** |
| --- | --- | --- | --- |
| Cholesterol (mmol/L) | 4,400 | 121,899 | 0.98 (0.95-1.01) |
| C-reactive protein (mg/L) | 4,394 | 121,701 | 1.04 (1.01-1.07) |
| Glycated haemoglobin (HbA1c) (mmol/mol) | 4,352 | 121,108 | 0.99 (0.96-1.02) |
| HDL-cholesterol (mmol/L) | 4,038 | 110,815 | 0.95 (0.92-0.98) |
| IGF-1 (nmol/L) | 4,365 | 121,223 | 1.05 (1.02-1.08) |
| SHBG (nmol/L) | 3,987 | 109,672 | 0.93 (0.90-0.96) |
| Testosterone (nmol/L) | 3,718 | 98,656 | 1.07 (1.05-1.09) |
| Total protein (g/L) | 4,038 | 110,757 | 0.97 (0.94-1.01) |
| Triglycerides (mmol/L) | 4,397 | 121,835 | 1.02 (0.99-1.05) |
| Albumin (g/L) | 4,041 | 110,861 | 0.95 (0.92-0.98) |
| Glucose (mmol/L) | 4,032 | 110,738 | 1.02 (0.99-1.05) |
| Alanine aminotransferase (U/L) | 4,400 | 121,904 | 1.03 (1.00-1.05) |
| Apolipoprotein A (g/L) | 3,994 | 109,646 | 0.96 (0.93-0.99) |
| Apolipoprotein B (g/L) | 4,386 | 121,539 | 1.00 (0.97-1.03) |
| Cystatin C (mg/L) | 4,396 | 121,887 | 1.03 (1.00-1.06) |
| Gamma-glutamyltransferase (U/L) | 4,396 | 121,858 | 1.03 (1.01-1.06) |
| Total bilirubin (umol/L) | 4,381 | 121,425 | 1.03 (1.00-1.06) |
| Urate (umol/L) | 4,393 | 121,761 | 1.06 (1.03-1.09) |

Hazard ratios per 1 standard deviation (SD) increment in each PC from Cox proportional hazards regression using age as the underlying time metric. Stratified by age in 5-year categories, center and diet, and adjusted for age alcohol frequency, smoking status, ethnicity, oral contraceptive, menopausal hormone treatment, physical activity, qualifications, townsend deprivation index and sedentary behavior.

HDL cholesterol: high-density lipoprotein cholesterol, IGF-1: insulin-like growth factor, SHBG: [sex hormone-binding globulin.](https://fr.wikipedia.org/wiki/Sex_hormone-binding_globulin)

**Supplementary Table 7:** Beta coefficient and P value of the association between principal components and biomarkers

|  |  | **PC1** | | | **PC2** | | **PC3** | | **PC4** | |
| --- | --- | --- | --- | --- | --- | --- | --- | --- | --- | --- |
| **Biomarkers** | n | Beta | P value | | Beta | P value | Beta | P value | Beta | P value |
| Cholesterol (mmol/L) | 121,899 | -0.039 | <0.001 | | -0.042 | <0.001 | 0.022 | <0.001 | -0.020 | <0.001 |
| C-reactive protein (mg/L) | 121,701 | 1.223 | <0.001 | | -0.262 | <0.001 | -0.159 | <0.001 | 0.074 | <0.001 |
| Glycated haemoglobin (HbA1c) (mmol/mol) | 121,108 | 1.319 | <0.001 | | -0.441 | <0.001 | 0.410 | <0.001 | 0.206 | <0.001 |
| HDL-cholesterol (mmol/L) | 110,815 | -0.131 | <0.001 | | 0.348 | <0.001 | -0.045 | <0.001 | -0.022 | <0.001 |
| IGF-1 (nmol/L) | 121,223 | -0.678 | <0.001 | | 0.199 | <0.001 | 0.214 | <0.001 | 0.004 | 0.780 |
| SHBG (nmol/L) | 109,672 | -12.899 | <0.001 | | 3.748 | 0.034 | -2.280 | <0.001 | -1.446 | <0.001 |
| Testosterone (nmol/L) | 98,656 | 0.050 | <0.001 | | -0.004 | 0.038 | -0.018 | <0.001 | 0.008 | <0.001 |
| Total protein (g/L) | 110,757 | 0.018 | 0.164 | | -0.201 | 0.032 | 0.110 | <0.001 | 0.042 | 0.001 |
| Triglycerides (mmol/L) | 121,835 | 0.261 | <0.001 | | -0.132 | <0.001 | 0.136 | <0.001 | 0.029 | <0.001 |
| Albumin (g/L) | 110,861 | -0.428 | <0.001 | | -0.065 | <0.001 | 0.177 | <0.001 | 0.045 | <0.001 |
| Glucose (mmol/L) | 110,738 | 0.167 | <0.001 | | -0.054 | <0.001 | 0.053 | <0.001 | 0.032 | <0.001 |
| Alanine aminotransferase (U/L) | 121,904 | 2.629 | <0.001 | | -1.162 | <0.001 | 0.816 | <0.001 | 0.386 | <0.001 |
| Apolipoprotein A (g/L) | 109,646 | -0.067 | <0.001 | | 0.012 | <0.001 | -0.021 | <0.001 | -0.013 | <0.001 |
| Apolipoprotein B (g/L) | 121,539 | 0.021 | <0.001 | | -0.019 | <0.001 | 0.014 | <0.001 | 0.001 | 0.087 |
| Cystatin C (mg/L) | 121,887 | 0.047 | 0.383 | | 0.000 | 0.461 | -0.004 | <0.001 | 0.004 | <0.001 |
| Gamma-glutamyltransferase (U/L) | 121,858 | 5.413 | <0.001 | | -2.822 | 0.094 | 1.448 | <0.001 | 0.246 | 0.014 |
| Total bilirubin (umol/L) | 121,425 | -0.280 | <0.001 | | 0.243 | <0.001 | 0.053 | <0.001 | 0.052 | <0.001 |
| Urate (umol/L) | 121,761 | 29.526 | | <0.001 | -5.707 | <0.001 | 0.777 | <0.001 | 4.069 | <0.001 |

HDL cholesterol: high-density lipoprotein cholesterol, IGF-1: insulin-like growth factor, SHBG: [sex hormone-binding globulin](https://fr.wikipedia.org/wiki/Sex_hormone-binding_globulin)

Models adjusted for age, center, diet, alcohol frequency, smoking status, ethnicity, oral contraceptive, menopausal hormone treatment, physical activity, qualifications, townsend deprivation index and sedentary behavior.

**Supplementary Table 8**: Beta coefficient (standard error) and P value for the four-way decomposition of each mediator of the associations between principal component 1 of body shape and postmenopausal breast cancer risk

|  | **TE** | | **CDE** | | **Intref** | | | **Intmed** | | **PIE** | |
| --- | --- | --- | --- | --- | --- | --- | --- | --- | --- | --- | --- |
| **Biomarkers** | Beta coef  (SE) | P value | Beta coef  (SE) | P value | Beta coef  (SE) | P value | Beta coef  (SE) | | P value | Beta coef  (SE) | P value |
| C-reactive protein (mg/L) | 0.168 (0.030) | <0.001 | 0.172 (0.031) | <0.001 | -0.007 (0.003) | 0.028 | -0.009 (0.004) | | 0.045 | 0.011 (0.006) | 0.008 |
| HDL-cholesterol (mmol/L) | 0.154 (0.030) | <0.001 | 0.150 (0.029) | <0.001 | 0.003 (0.004) | 0.425 | -0.017 (0.002) | | 0.418 | 0.003 (0.004) | 0.532 |
| IGF-1 (nmol/L) | 0.143 (0.029) | <0.001 | 0.146 (0.029) | <0.001 | 0.015 (0.005) | 0.003 | -0.012 (0.004) | | 0.003 | -0.006 (0.005) | 0.205 |
| SHBG (nmol/L) | 0.170 (0.029) | <0.001 | 0.150 (0.029) | <0.001 | 0.009 (0.012) | 0.458 | -0.009 (0.012) | | 0.449 | 0.017 (0.009) | 0.069 |
| Testosterone (nmol/L) | 0.151 (0.029) | <0.001 | 0.134 (0.029) | <0.001 | -0.0006 (0.005) | 0.233 | 0.002 (0.003) | | 0.413 | 0.015 (0.003) | <0.001 |
| Triglycerides (mmol/L) | 0.145 (0.029) | <0.001 | 0.148 (0.030) | <0.001 | -0.002 (0.004) | 0.616 | -0.0004 (0.001) | | 0.613 | -0.0005 (0.001) | 0.658 |
| Albumin (g/L) | 0.162 (0.030) | <0.001 | 0.151 (0.030) | <0.001 | 0.001 (0.003) | 0.591 | -0.003 (0.004) | | 0.548 | 0.012 (0.005) | 0.022 |
| Glucose (mmol/L) | 0.147 (0.030) | <0.001 | 0.148 (0.030) | <0.001 | -0.001 (0.001) | 0.422 | -0.002 (0.002) | | 0.398 | 0.001 (0.003) | 0.629 |
| Alanine Aminotransferase (U/L) | 0.146 (0.029) | <0.001 | 0.146 (0.030) | <0.001 | 0.0000 (0.0003) | 0.933 | -0.0003 (0.004) | | 0.947 | 0.0002 (0.004) | 0.950 |
| Apolipoprotein A (g/L) | 0.149 (0.029) | <0.001 | 0.146 (0.029) | <0.001 | 0.012 (0.002) | 0.475 | 0.0008 (0.001) | | 0.463 | 0.0007 (0.003) | 0.815 |
| Gamma-Glutamyltransferase (U/L) | 0.146 (0.029) | <0.001 | 0.148 (0.030) | <0.001 | -0.003 (0.005) | 0.566 | -0.00001 (0.0002) | | 0.749 | 0.0001 (0.0002) | 0.512 |
| Total bilirubin (umol/L) | 0.143 (0.029) | <0.001 | 0.142 (0.030) | <0.001 | 0.003 (0.007) | 0.687 | -0.0005 (0.001) | | 0.682 | -0.002 (0.001) | 0.103 |
| Urate (umol/L) | 0.144 (0.032) | <0.001 | 0.149 (0.032) | <0.001 | 0.001 (0.003) | 0.629 | -0.004 (0.009) | | 0.637 | -0.001 (0.009) | 0.873 |

TE=total effect (total excess relative risk); CDE=excess relative risk due to controlled direct effect; INTref=excess relative risk due to reference interaction; INTmed=excess relative risk due to mediated interaction; PIE=excess relative risk due to pure indirect effect. Output of mediation analysis with causal effects estimated for a change in PC from the 25th to the 75th percentile. Controlled direct effects are computed fixing the mediators at their median levels.

HDL cholesterol: High-density lipoprotein cholesterol, IGF-1: insulin-like growth factor, SHBG: Sex hormone-binding globulin.

Models adjusted for age, center, diet, alcohol frequency, smoking status, ethnicity, oral contraceptive, menopausal hormone treatment, physical activity, qualifications, townsend deprivation index and sedentary behavior, and mutually adjusted for biomarkers.

**Supplementary Table 9**: Beta coefficient (standard error) and P value for the four-way decomposition of each mediator of the associations between principal component 2 of body shape and postmenopausal breast cancer risk

|  | **TE** | | **CDE** | | **Intref** | | | **Intmed** | | **PIE** | |
| --- | --- | --- | --- | --- | --- | --- | --- | --- | --- | --- | --- |
| **Biomarkers** | Beta coef  (SE) | P value | Beta coef  (SE) | P value | Beta coef  (SE) | P value | Beta coef  (SE) | | P value | Beta coef  (SE) | P value |
| C-reactive protein (mg/L) | 0.127 (0.029) | <0.001 | 0.125 (0.029) | <0.001 | 0.0031 (0.008) | 0.682 | -0.0002 (0.001) | | 0.682 | -0.0006 (0.001) | 0.221 |
| HDL-cholesterol (mmol/L) | 0.128 (0.029) | <0.001 | 0.126 (0.029) | <0.001 | 0.0026 (0.002) | 0.253 | 0.0007 (0.001) | | 0.251 | -0.0022 (0.001) | 0.109 |
| IGF-1 (nmol/L) | 0.130 (0.029) | <0.001 | 0.132 (0.029) | <0.001 | -0.0039 0.003) | 0.158 | -0.0017 (0.001) | | 0.173 | 0.0036 (0.001) | 0.002 |
| SHBG (nmol/L) | 0.123 (0.029) | <0.001 | 0.123 (0.029) | <0.001 | 0.0044 (0.002) | 0.051 | 0.0037 (0.002) | | 0.024 | -0.0075 (0.002) | 0.000 |
| Testosterone (nmol/L) | 0.127 (0.029) | <0.001 | 0.128 (0.029) | <0.001 | -0.0002 (0.003) | 0.936 | -0.0001 (0.0002) | | 0.841 | -0.0009 (0.001) | 0.114 |
| Triglycerides (mmol/L) | 0.130 (0.029) | <0.001 | 0.134 (0.030) | <0.001 | -0.0066 (0.005) | 0.190 | 0.0019 (0.002) | | 0.211 | 0.0002 (0.001) | 0.882 |
| Albumin (g/L) | 0.131 (0.029) | <0.001 | 0.128 (0.029) | <0.001 | -0.0004 (0.001) | 0.656 | 0.0004 (0.0003) | | 0.731 | 0.0029 (0.001) | 0.041 |
| Glucose (mmol/L) | 0.128 (0.029) | <0.001 | 0.130 (0.029) | <0.001 | -0.0018 (0.002) | 0.458 | 0.0002 (0.003) | | 0.521 | -0.0001 (0.0002) | 0.638 |
| Alanine Aminotransferase (U/L) | 0.127 (0.029) | <0.001 | 0.129 (0.029) | <0.001 | -0.0004 (0.003) | 0.886 | 0.0001 (0.001) | | 0.892 | -0.0008 (0.001) | 0.455 |
| Apolipoprotein A (g/L) | 0.129 (0.029) | <0.001 | 0.124 (0.029) | <0.001 | 0.0062 (0.003) | 0.058 | -0.0012 (0.001) | | 0.058 | -0.0005 (0.001) | 0.644 |
| Gamma-Glutamyltransferase (U/L) | 0.127 (0.029) | <0.001 | 0.129 (0.029) | <0.001 | -0.0011 (0.005) | 0.817 | 0.0001 (0.001) | | 0.818 | -0.0007 (0.001) | 0.303 |
| Total bilirubin (umol/L) | 0.130 (0.029) | <0.001 | 0.127 (0.030) | <0.001 | 0.0008 (0.005) | 0.880 | 0.0003 (0.002) | | 0.874 | 0.0019 (0.001) | 0.250 |
| Urate (umol/L) | 0.128 (0.029) | <0.001 | 0.129 (0.029) | <0.001 | -0.0006 (0.003) | 0.827 | 0.00002 (0.0001) | | 0.874 | -0.0001 (0.0001) | 0.545 |

TE=total effect (total excess relative risk); CDE=excess relative risk due to controlled direct effect; INTref=excess relative risk due to reference interaction; INTmed=excess relative risk due to mediated interaction; PIE=excess relative risk due to pure indirect effect. Output of mediation analysis with causal effects estimated for a change in PC from the 25th to the 75th percentile. Controlled direct effects are computed fixing the mediators at their median levels.

HDL cholesterol: High-density lipoprotein cholesterol, IGF-1: insulin-like growth factor, SHBG: Sex hormone-binding globulin.

Models adjusted for age, center, diet, alcohol frequency, smoking status, ethnicity, oral contraceptive, menopausal hormone treatment, physical activity, qualifications, townsend deprivation index and sedentary behavior, and mutually adjusted for biomarkers

**IGF-1**

**
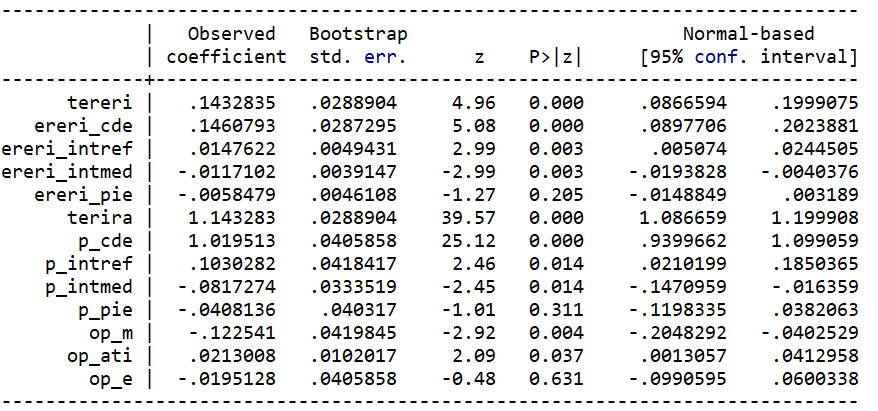
**

**Testosterone**

**
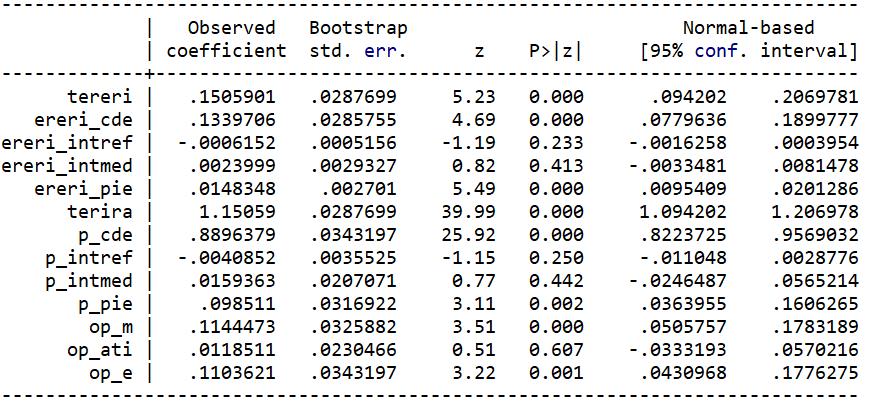
**

tereri=total excess relative risk; ereri_cde=excess relative risk due to controlled direct effect; ereri_intref=excess relative risk due to reference interaction; ereri_intmed=excess relative risk due to mediated interaction; ereri_pie=excess relative risk due to pure indirect effect; terira=total effect risk ratio; p_cde=proportion controlled direct effect; p_intref=proportion reference interaction; p_intmed=proportion mediated interaction; p_pie=proportion pure indirect effect; op_m=overall proportion mediated; op_ati=overall proportion attributable to interaction; op_e=overall proportion eliminated.

**Supplementary Figure 12: Full output of the 4-way decomposition mediation analysis with IGF-1 and testosterone as mediators, PC1 as exposure and breast cancer as outcome.** Causal effects were estimated for a change in PC1 from the 25th to the 75th percentile. Controlled direct effects are computed fixing the mediators at their median levels.

Models adjusted for age, center, diet, alcohol frequency, smoking status, ethnicity, oral contraceptive, menopausal hormone treatment, physical activity, qualifications, townsend deprivation index and sedentary behavior, and mutually adjusted for biomarkers.

IGF-1: insulin-like growth factor.

**IGF-1**

**
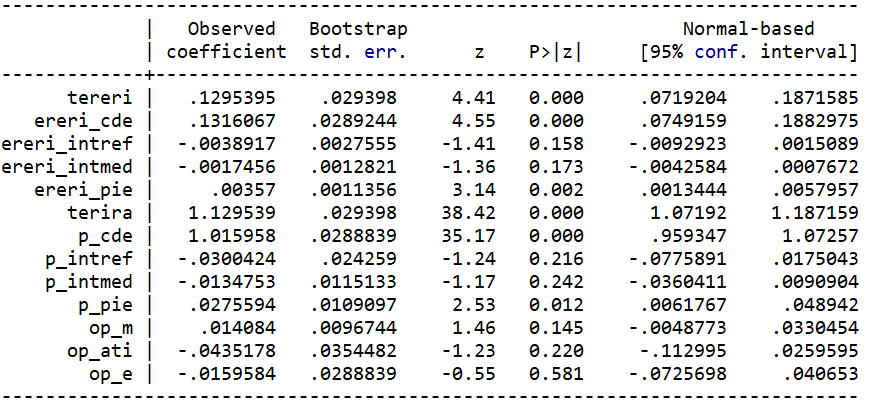
**

**SHBG**


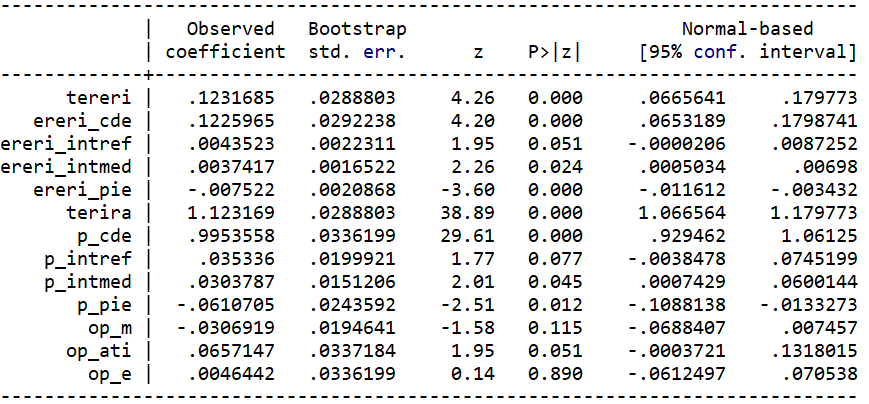


tereri=total excess relative risk; ereri_cde=excess relative risk due to controlled direct effect; ereri_intref=excess relative risk due to reference interaction; ereri_intmed=excess relative risk due to mediated interaction; ereri_pie=excess relative risk due to pure indirect effect; terira=total effect risk ratio; p_cde=proportion controlled direct effect; p_intref=proportion reference interaction; p_intmed=proportion mediated interaction; p_pie=proportion pure indirect effect; op_m=overall proportion mediated; op_ati=overall proportion attributable to interaction; op_e=overall proportion eliminated.

**Supplementary Figure 13: Full output of the 4-way decomposition mediation analysis with IGF-1 and SHBG and as mediators, PC2 as exposure and breast cancer as outcome.** Causal effects were estimated for a change in PC2 from the 25th to the 75th percentile. Controlled direct effects are computed fixing the mediators at their median levels.

Models adjusted for age, center, diet, alcohol frequency, smoking status, ethnicity, oral contraceptive, menopausal hormone treatment, physical activity, qualifications, townsend deprivation index and sedentary behavior, and mutually adjusted for biomarkers.

IGF-1: insulin-like growth factor, SHBG: Sex hormone-binding globulin.

**Supplementary Table 10** Beta coefficient (standard error) and P value for the four-way decomposition of each mediator of the associations between principal component 1 of body shape and postmenopausal breast cancer risk

|  | **TE** | | **CDE** | | **Intref** | | | **Intmed** | | **PIE** | |
| --- | --- | --- | --- | --- | --- | --- | --- | --- | --- | --- | --- |
| **Biomarkers** | Beta coef  (SE) | P value | Beta coef  (SE) | P value | Beta coef  (SE) | P value | Beta coef  (SE) | | P value | Beta coef  (SE) | P value |
| C-reactive protein (mg/L) | 0.185 (0.021) | <0.001 | 0.191 (0.024) | <0.001 | -0.005 (0.002) | 0.006 | -0.016 (0.006) | | 0.009 | 0.015 (0.006) | 0.013 |
| HDL-cholesterol (mmol/L) | 0.182 (0.026) | <0.001 | 0.173 (0.027) | <0.001 | 0.018 (0.009) | 0.040 | -0.022 (0.010) | | 0.034 | 0.012 (0.009) | 0.170 |
| IGF-1 (nmol/L) | 0.173 (0.026) | <0.001 | 0.178 (0.025) | <0.001 | 0.010 (0.004) | 0.014 | -0.007 (0.003) | | 0.011 | -0.007 (0.003) | 0.019 |
| SHBG (nmol/L) | 0.178 (0.024) | <0.001 | 0.155 (0.025) | <0.001 | 0.023 (0.011) | 0.045 | -0.026 (0.013) | | 0.037 | 0.027 (0.012) | 0.023 |
| Testosterone (nmol/L) | 0.162 (0.026) | <0.001 | 0.147 (0.027) | <0.001 | -0.001 (0.001) | 0.339 | 0.001 (0.002) | | 0.519 | 0.014 (0.002) | <0.001 |
| Triglycerides (mmol/L) | 0.170 (0.021) | <0.001 | 0.178 (0.022) | <0.001 | 0.0004 (0.001) | 0.447 | -0.010 (0.007) | | 0.162 | 0.002 (0.009) | 0.822 |
| Albumin (g/L) | 0.176 (0.025) | <0.001 | 0.169 (0.025) | <0.001 | 0.003 (0.002) | 0.116 | -0.007 (0.004) | | 0.075 | 0.011 (0.004) | 0.008 |
| Glucose (mmol/L) | 0.172 (0.024) | <0.001 | 0.173 (0.025 | <0.001 | -0.001 (0.001) | 0.293 | -0.004 (0.003) | | 0.134 | 0.005 (0.005) | 0.303 |
| Alanine Aminotransferase (U/L) | 0.166 (0.023) | <0.001 | 0.165 (0.024) | <0.001 | 0.0005 (0.001) | 0.725 | -0.002 (0.005) | | 0.738 | 0.003 (0.006) | 0.609 |
| Apolipoprotein A (g/L) | 0.177 (0.025) | <0.001 | 0.169 (0.025) | <0.001 | 0.010 (0.006) | 0.121 | -0.012 (0.007) | | 0.114 | 0.010 (0.006) | 0.113 |
| Gamma-Glutamyltransferase (U/L) | 0.167 (0.023) | <0.001 | 0.165 (0.023) | <0.001 | -0.002 (0.003) | 0.394 | -0.002 (0.003) | | 0.414 | 0.006 (0.004) | 0.074 |
| Total bilirubin (umol/L) | 0.166 (0.023) | <0.001 | 0.166 (0.024) | <0.001 | 0.005 (0.008) | 0.484 | -0.002 (0.002) | | 0.474 | -0.003 (0.001) | 0.043 |
| Urate (umol/L) | 0.176 (0.024) | <0.001 | 0.167 (0.026) | <0.001 | 0.007 (0.006) | 0.212 | -0.013 (0.011) | | 0.208 | 0.016 (0.011) | 0.160 |

TE=total effect (total excess relative risk); CDE=excess relative risk due to controlled direct effect; INTref=excess relative risk due to reference interaction; INTmed=excess relative risk due to mediated interaction; PIE=excess relative risk due to pure indirect effect. Output of mediation analysis with causal effects estimated for a change in PC1 from the 25th to the 75th percentile. Controlled direct effects are computed fixing the mediators at their median levels

HDL cholesterol: high-density lipoprotein cholesterol, IGF-1: insulin-like growth factor, SHBG: sex hormone-binding globulin.

M Models adjusted for age, center, diet, alcohol frequency, smoking status, ethnicity, oral contraceptive, menopausal hormone treatment, physical activity, qualifications, townsend deprivation index and sedentary behavior

**Supplementary** **Table 11:** Proportion attributable for the four-way decomposition of each mediator of the associations between principal component 1 of body shape and postmenopausal breast cancer risk

|  | **P_ CDE** | | **P_ INTref** | | **P_INTmed** | | **P_PIE** | | **OP_M** | |
| --- | --- | --- | --- | --- | --- | --- | --- | --- | --- | --- |
| **Biomarkers** | **Proportion** | **P value** | **Proportion** | **P value** | **Proportion** | **P value** | **Proportion** | **P value** | **Proportion** | **P value** |
| C-reactive protein (mg/L) | 103.4% | <0.001 | -2.7% | 0.003 | -8.6% | 0.006 | 8.0% | 0.009 | -0.6% | 0.876 |
| HDL-cholesterol (mmol/L) | 94.9% | <0.001 | 10.1% | 0.036 | -11.9% | 0.030 | 6.8% | 0.201 | -5.0% | 0.447 |
| IGF1 (nmol/L) | 102.8% | <0.001 | 5.6% | 0.014 | -4.3% | 0.010 | -4.1% | 0.054 | -8.4% | <0.001 |
| SHBG (nmol/L) | 87.2% | <0.001 | 12.7% | 0.050 | -14.8% | 0.042 | 15.0% | 0.033 | 0.2% | 0.983 |
| Testosterone (nmol/L) | 90.7% | <0.001 | -0.4% | 0.342 | 0.8% | 0.538 | 8.9% | <0.001 | 9.7% | <0.001 |
| Triglycerides (mmol/L) | 104.9% | <0.001 | -0.3% | 0.443 | -5.9% | 0.183 | 1.2% | 0.828 | -4.7% | 0.336 |
| Albumin (g/L) | 95.7% | <0.001 | 1.8% | 0.127 | -3.9% | 0.089 | 6.5% | 0.011 | 2.6% | 0.312 |
| Glucose (mmol/L) | 100.3% | <0.001 | -0.7% | 0.298 | -2.5% | 0.143 | 2.9% | 0.323 | 0.3% | 0.885 |
| Alanine aminotransferase (U/L) | 99.5% | <0.001 | -0.3% | 0.734 | -0.9% | 0.746 | 1.7% | 0.634 | 0.8% | 0.828 |
| Apolipoprotein A (g/L) | 95.2% | <0.001 | 5.6% | 0.113 | -6.6% | 0.107 | 5.8% | 0.109 | -0.8% | 0.853 |
| Gamma-glutamyltransferase (U/L) | 99.0% | <0.001 | -1.4% | 0.414 | -1.5% | 0.428 | 3.9% | 0.094 | 2.4% | 0.228 |
| Total bilirubin (umol/L) | 99.6% | <0.001 | 3.2% | 0.507 | -0.9% | 0.499 | -1.8% | 0.049 | -2.7% | 0.053 |
| Urate (umol/L) | 94.7% | <0.001 | 3.9% | 0.216 | -7.5% | 0.212 | 8.9% | 0.185 | 1.4% | 0.844 |

P_CDE=proportion of controlled direct effect. P_INTref=proportion of reference interaction. P_INTmed=proportion of mediated interaction. P_PIE=proportion of pure indirect effect. OP_M=overall proportion mediated. Output of mediation analysis with causal effects estimated for a change in PC1 from the 25th to the 75th percentile. Controlled direct effects are computed fixing the mediators at their median levels.

HDL cholesterol: high-density lipoprotein cholesterol, IGF-1: insulin-like growth factor, SHBG: sex hormone-binding globulin.

Models adjusted for age, center, diet, alcohol frequency, smoking status, ethnicity, oral contraceptive, menopausal hormone treatment, physical activity, qualifications, townsend deprivation index and sedentary behavior

**Supplementary Table 12:** Beta coefficient (standard error) and P value for the four-way decomposition of each mediator of the associations between principal component 2 of body shape and postmenopausal breast cancer risk

|  | **TE** | | **CDE** | | **Intref** | | | **Intmed** | | **PIE** | |
| --- | --- | --- | --- | --- | --- | --- | --- | --- | --- | --- | --- |
| **Biomarkers** | Beta coef  (SE) | P value | Beta coef  (SE) | P value | Beta coef  (SE) | P value | Beta coef  (SE) | | P value | Beta coef  (SE) | P value |
| C-reactive protein (mg/L) | 0.11 (0.022) | <0.001 | 0.11 (0.022) | <0.001 | 0.004 (0.005) | 0.487 | -0.001 (0.001) | | 0.482 | -0.002 (0.001) | 0.038 |
| HDL-cholesterol (mmol/L) | 0.11 (0.025) | <0.001 | 0.12 (0.025) | <0.001 | 0.001 (0.001) | 0.397 | 0.003 (0.002) | | 0.172 | -0.009 (0.002) | 0.001 |
| IGF-1 (nmol/L) | 0.11 (0.026) | <0.001 | 0.11 (0.026) | <0.001 | -0.002 (0.002) | 0.382 | -0.001 (0.001) | | 0.428 | 0.003 (0.001) | 0.001 |
| SHBG (nmol/L) | 0.11 (0.026) | <0.001 | 0.12 (0.602) | <0.001 | 0.001 (0.001) | 0.429 | 0.009 (0.003) | | 0.009 | -0.018 (0.003) | <0.001 |
| Testosterone (nmol/L) | 0.11 (0.027) | <0.001 | 0.11 (0.027) | <0.001 | -0.001 (0.002) | 0.823 | 0.00002 (0.0001) | | 0.889 | -0.001 (0.001) | 0.174 |
| Triglycerides (mmol/L) | 0.11 (0.026) | <0.001 | 0.12 (0.026) | <0.001 | -0.013 (0.006) | 0.032 | 0.007 (0.003) | | 0.035 | -0.009 (0.003) | 0.007 |
| Albumin (g/L) | 0.12 (0.025) | <0.001 | 0.11 (0.025) | <0.001 | 0.0001 (0.0003) | 0.781 | 0.0005 (0.001) | | 0.572 | 0.002 (0.001) | 0.017 |
| Glucose (mmol/L) | 0.12 (0.023) | <0.001 | 0.12 (0.023) | <0.001 | -0.001 (0.003) | 0.615 | 0.0004 (0.001) | | 0.620 | -0.001 (0.001) | 0.128 |
| Alanine aminotransferase (U/L) | 0.11 (0.024) | <0.001 | 0.12 (0.024) | <0.001 | -0.002 (0.005) | 0.700 | 0.001 (0.002) | | 0.715 | -0.004 (0.002) | 0.042 |
| Apolipoprotein A (g/L) | 0.11 (0.023) | <0.001 | 0.11 (0.023) | <0.001 | 0.001 (0.001) | 0.344 | 0.001 (0.001) | | 0.207 | -0.003 (0.001) | 0.002 |
| Gamma-glutamyltransferase (U/L) | 0.11 (0.024) | <0.001 | 0.11 (0.024) | <0.001 | 0.003 (0.005) | 0.592 | -0.001 (0.002) | | 0.584 | -0.004 (0.001) | 0.002 |
| Total bilirubin (umol/L) | 0.11 (0.022) | <0.001 | 0.10 (0.023) | <0.001 | 0.004 (0.004) | 0.420 | 0.001 (0.002) | | 0.414 | 0.001 (0.002) | 0.461 |
| Urate (umol/L) | 0.11 (0.021) | <0.001 | 0.12 (0.021) | <0.001 | -0.001 (0.003) | 0.871 | 0.000 (0.002) | | 0.916 | -0.006 (0.002) | <0.001 |

TE=total effect (total excess relative risk); CDE=excess relative risk due to controlled direct effect; INTref=excess relative risk due to reference interaction; INTmed=excess relative risk due to mediated interaction; PIE=excess relative risk due to pure indirect effect. Output of mediation analysis with causal effects estimated for a change in PC2 from the 25th to the 75th percentile. Controlled direct effects are computed fixing the mediators at their median levels

HDL cholesterol: high-density lipoprotein cholesterol, IGF-1: insulin-like growth factor, SHBG: sex hormone-binding globulin.

Models adjusted for age, center, diet, alcohol frequency, smoking status, ethnicity, oral contraceptive, menopausal hormone treatment, physical activity, qualifications, townsend deprivation index and sedentary behavior

**Supplementary Table 13:** Proportion attributable for the four-way decomposition of each mediator of the associations between principal component 2 of body shape and postmenopausal breast cancer risk

|  | **P_ CDE** | | **P_ INTref** | | **P_INTmed** | | **P_PIE** | | **OP_M** | |
| --- | --- | --- | --- | --- | --- | --- | --- | --- | --- | --- |
| **Biomarkers** | **Proportion** | **P value** | **Proportion** | **P value** | **Proportion** | **P value** | **Proportion** | **P value** | **Proportion** | **P value** |
| C-reactive protein (mg/L) | 99.7% | <0.001 | 3.2% | 0.475 | -0.7% | 0.469 | -2.3% | 0.061 | -3.0% | 0.011 |
| HDL-cholesterol (mmol/L) | 104.1% | <0.001 | 0.6% | 0.429 | 2.9% | 0.234 | -7.6% | 0.010 | -4.7% | 0.024 |
| IGF1 (nmol/L) | 99.6% | <0.001 | -1.6% | 0.487 | -0.7% | 0.492 | 2.7% | 0.039 | 2.0% | 0.067 |
| SHBG (nmol/L) | 107.6% | <0.001 | 1.1% | 0.447 | 7.5% | 0.026 | -16.2% | <0.001 | -8.7% | 0.018 |
| Testosterone (nmol/L) | 101.3% | <0.001 | -0.5% | 0.837 | 0.0% | 0.896 | -0.8% | 0.240 | -0.8% | 0.217 |
| Triglycerides (mmol/L) | 113.7% | <0.001 | -12.2% | 0.067 | 6.8% | 0.068 | -8.3% | 0.054 | -1.5% | 0.712 |
| Albumin (g/L) | 98.0% | <0.001 | -0.1% | 0.805 | 0.4% | 0.611 | 1.7% | 0.051 | 2.1% | 0.040 |
| Glucose (mmol/L) | 102.1% | <0.001 | -1.3% | 0.650 | 0.4% | 0.657 | -1.3% | 0.184 | -0.9% | 0.510 |
| Alanine aminotransferase (U/L) | 104.6% | <0.001 | -1.6% | 0.726 | 0.6% | 0.739 | -3.5% | 0.099 | -2.9% | 0.191 |
| Apolipoprotein A (g/L) | 100.7% | <0.001 | 0.9% | 0.366 | 1.3% | 0.247 | -3.0% | 0.019 | -1.7% | 0.145 |
| Gamma-glutamyltransferase (U/L) | 101.8% | <0.001 | 2.6% | 0.651 | -0.8% | 0.641 | -3.6% | 0.034 | -4.4% | 0.063 |
| Total bilirubin (umol/L) | 94.1% | <0.001 | 3.2% | 0.470 | 1.4% | 0.464 | 1.3% | 0.516 | 2.7% | 0.096 |
| Urate (umol/L) | 106.1% | <0.001 | -0.5% | 0.880 | 0.2% | 0.921 | -5.8% | 0.012 | -5.6% | 0.011 |

P_CDE=proportion of controlled direct effect. P_INTref=proportion of reference interaction. P_INTmed=proportion of mediated interaction. P_PIE=proportion of pure indirect effect. OP_M=overall proportion mediated. Output of mediation analysis with causal effects estimated for a change in PC2 from the 25th to the 75th percentile. Controlled direct effects are computed fixing the mediators at their median levels.

HDL cholesterol: high-density lipoprotein cholesterol, IGF-1: insulin-like growth factor, SHBG: sex hormone-binding globulin.

Models adjusted for age, center, diet, alcohol frequency, smoking status, ethnicity, oral contraceptive, menopausal hormone treatment, physical activity, qualifications, townsend deprivation index and sedentary behavior

**Testosterone**


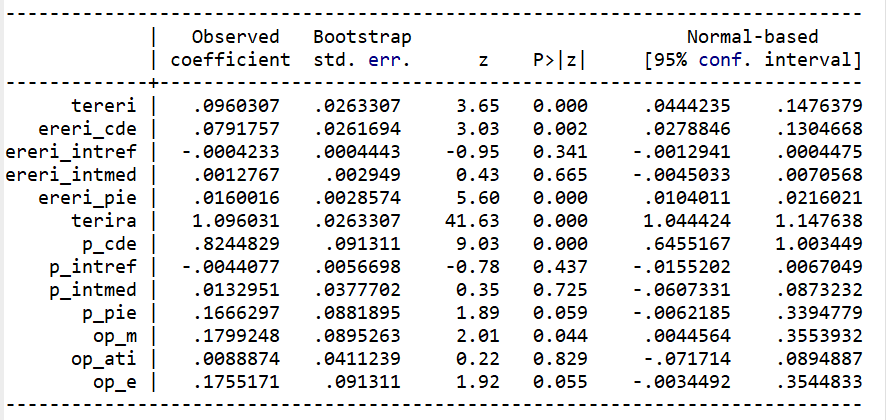
tereri=total excess relative risk; ereri_cde=excess relative risk due to controlled direct effect; ereri_intref=excess relative risk due to reference interaction; ereri_intmed=excess relative risk due to mediated interaction; ereri_pie=excess relative risk due to pure indirect effect; terira=total effect risk ratio; p_cde=proportion controlled direct effect; p_intref=proportion reference interaction; p_intmed=proportion mediated interaction; p_pie=proportion pure indirect effect; op_m=overall proportion mediated; op_ati=overall proportion attributable to interaction; op_e=overall proportion eliminated.

**Supplementary Figure 14: Full output of the 4-way decomposition mediation analysis with testosterone as mediators, BMI as exposure and breast cancer as outcome.** Causal effects were estimated for a change in BMI from the 25th to the 75th percentile. Controlled direct effects are computed fixing the mediators at their median levels.

Models adjusted for age, center, diet, alcohol frequency, smoking status, ethnicity, oral contraceptive, menopausal hormone treatment, physical activity, qualifications, townsend deprivation index and sedentary behavior, and mutually adjusted for biomarkers.

IGF-1: insulin-like growth factor, BMI: body mass index.

**C-reactive protein**


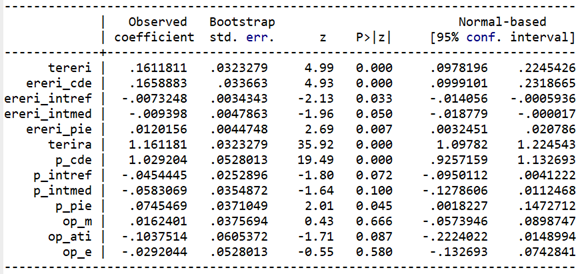


**IGF-1**


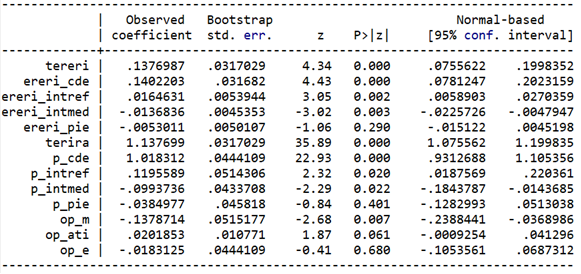


**Testosterone**


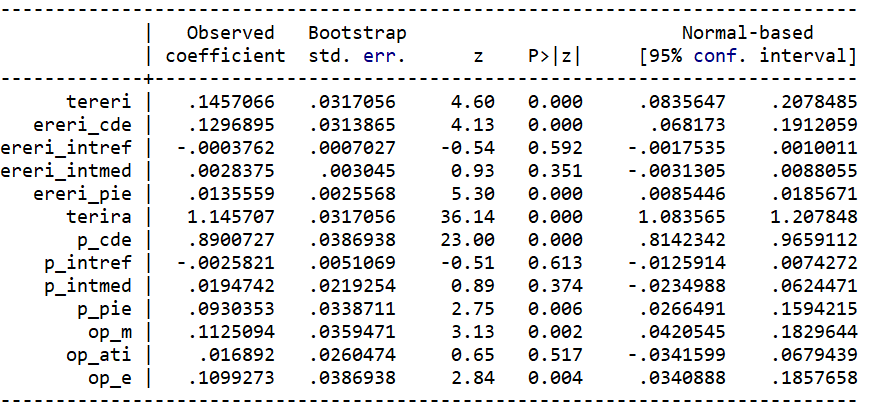


**Albumin**


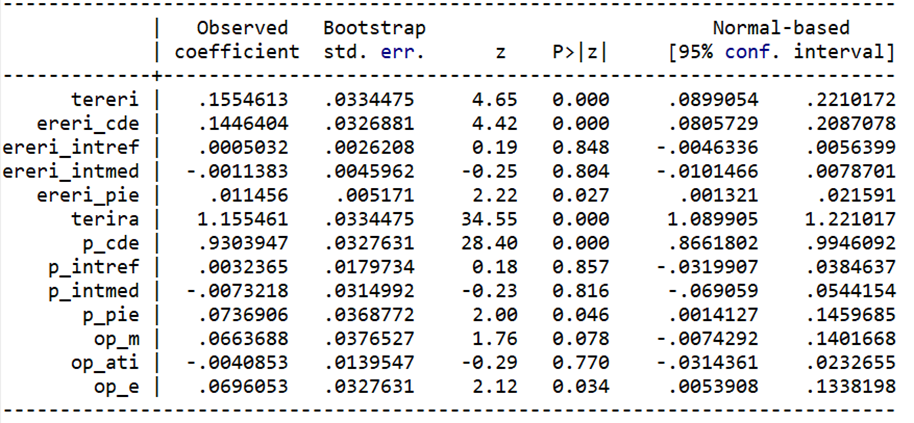


tereri=total excess relative risk; ereri_cde=excess relative risk due to controlled direct effect; ereri_intref=excess relative risk due to reference interaction; ereri_intmed=excess relative risk due to mediated interaction; ereri_pie=excess relative risk due to pure indirect effect; terira=total effect risk ratio; p_cde=proportion controlled direct effect; p_intref=proportion reference interaction; p_intmed=proportion mediated interaction; p_pie=proportion pure indirect effect; op_m=overall proportion mediated; op_ati=overall proportion attributable to interaction; op_e=overall proportion eliminated.

**Supplementary Figure 15: Full output of the 4-way decomposition mediation analysis with C-reactive protein, IGF-1 and testosterone as mediators, waist circumference as exposure and breast cancer as outcome.** Causal effects were estimated for a change in waist circumference from the 25th to the 75th percentile. Controlled direct effects are computed fixing the mediators at their median levels.

Models adjusted for age, center, diet, alcohol frequency, smoking status, ethnicity, oral contraceptive, menopausal hormone treatment, physical activity, qualifications, townsend deprivation index and sedentary behavior, and mutually adjusted for biomarkers.

IGF-1: insulin-like growth factor.

**IGF1**


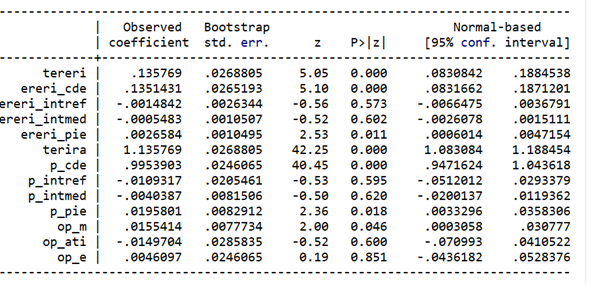


**SHGB**


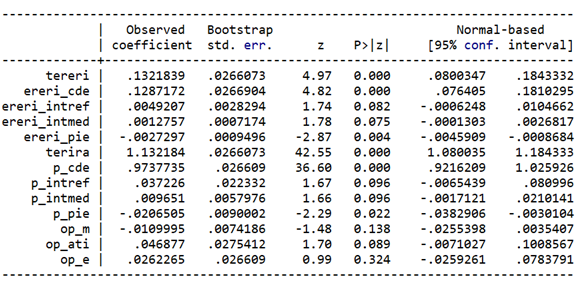


**Testosterone**


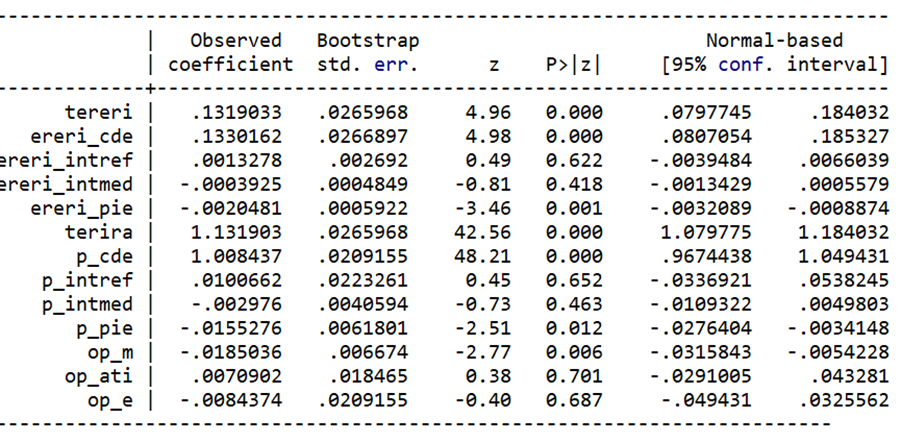


**Albumin**


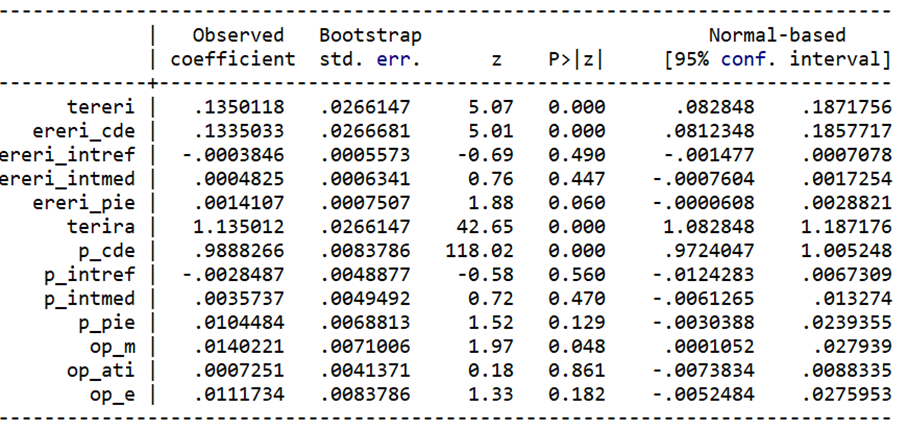


tereri=total excess relative risk; ereri_cde=excess relative risk due to controlled direct effect; ereri_intref=excess relative risk due to reference interaction; ereri_intmed=excess relative risk due to mediated interaction; ereri_pie=excess relative risk due to pure indirect effect; terira=total effect risk ratio; p_cde=proportion controlled direct effect; p_intref=proportion reference interaction; p_intmed=proportion mediated interaction; p_pie=proportion pure indirect effect; op_m=overall proportion mediated; op_ati=overall proportion attributable to interaction; op_e=overall proportion eliminated.

**Supplementary Figure 16: Full output of the 4-way decomposition mediation analysis with C-reactive protein, IGF-1 and testosterone as mediators, height as exposure and breast cancer as outcome.** Causal effects were estimated for a change in height from the 25th to the 75th percentile. Controlled direct effects are computed fixing the mediators at their median levels.

Models adjusted for age, center, diet, alcohol frequency, smoking status, ethnicity, oral contraceptive, menopausal hormone treatment, physical activity, qualifications, townsend deprivation index and sedentary behavior, and mutually adjusted for biomarkers.

IGF-1: insulin-like growth factor.
